# The secretome of irradiated peripheral blood mononuclear cells attenuates activation of mast cells and basophils

**DOI:** 10.1016/j.ebiom.2022.104093

**Published:** 2022-06-04

**Authors:** Maria Laggner, Gabriela Sánchez Acosta, Claudia Kitzmüller, Dragan Copic, Florian Gruber, Lukas Matthäus Altenburger, Vera Vorstandlechner, Alfred Gugerell, Martin Direder, Katharina Klas, Daniel Bormann, Anja Peterbauer, Akira Shibuya, Barbara Bohle, Hendrik Jan Ankersmit, Michael Mildner

**Affiliations:** a Department of Thoracic Surgery, Medical University of Vienna, Waehringer Guertel 18-20, 1090 Vienna, Austria; b Laboratory for Cardiac and Thoracic Diagnosis and Regeneration, Vienna, Austria; c Department of Pathophysiology and Allergy Research, Medical University of Vienna, Vienna, Austria; d Aposcience AG, Vienna, Austria; e Department of Dermatology, Medical University of Vienna, Lazarettgasse 14, 1090 Vienna, Austria; f Department of Plastic, Reconstructive and Aesthetic Surgery, Medical University of Vienna, Vienna, Austria; g Austrian Red Cross Blood Transfusion Service of Upper Austria, Linz, Austria; h Department of Immunology, Faculty of Medicine, University of Tsukuba, Tsukuba, Japan

**Keywords:** Anti-allergic therapeutic secretome, Basophil activation, Birch pollen allergy, Mast cell degranulation

## Abstract

**Background:**

IgE-mediated hypersensitivity is becoming increasingly prevalent and activation of mast cells and basophils represent key events in the pathophysiology of allergy. We have previously reported that the secretome of γ-irradiated peripheral blood mononuclear cells (PBMCsec) exerts beneficial anti-inflammatory effects. Yet, its ability to alleviate allergic symptoms has not been investigated so far.

**Methods:**

Several experimental *in vitro* and *in vivo* models have been used in this basic research study. A murine ear swelling model was used to study the effects of PBMCsec on 48/80-induced mast cell degranulation *in vivo*. The transcriptional profile of murine mast cells was analysed by single cell RNA sequencing (scRNAseq). Mast cell activation was studied *in vitro* using primary skin mast cells. Basophils from individuals allergic to birch pollens were used to investigate basophile activation by allergens. Transcriptomic and lipidomic analyses were used to identify mRNA expression and lipid species present in PBMCsec, respectively.

**Findings:**

Topical application of PBMCsec on mouse ears (C57BL/6) significantly reduced tissue swelling following intradermal injection of compound 48/80, an inducer of mast cell degranulation. Single cell RNA sequencing of PBMCsec-treated murine dermal mast cells (Balb/c) revealed a downregulation of genes involved in immune cell degranulation and Fc-receptor signalling. In addition, treatment of primary human dermal mast cells with PBMCsec strongly inhibited compound 48/80- and α-IgE-induced mediator release *in vitro*. Furthermore, PBMCsec remarkably attenuated allergen driven activation of basophils from allergic individuals. Transcriptomic analysis of these basophils showed that PBMCsec downregulated a distinct gene battery involved in immune cell degranulation and Fc-receptor signalling, corroborating results obtained from dermal mast cells. Finally, we identified the lipid fraction of PBMCsec as the major active ingredient involved in effector cell inhibition.

**Interpretation:**

Collectively, our data demonstrate that PBMCsec is able to reduce activation of mast cells and basophils, encouraging further studies on the potential use of PBMCsec for treating allergy.

**Funding:**

Austrian Research Promotion Agency (852748 and 862068, 2015-2019), Vienna Business Agency (2343727, 2018-2020), Aposcience AG, Austrian Federal Ministry of Education, Science and Research (SPA06/055), Danube Allergy Research Cluster, Austrian Science Fund (I4437 and P32953).

**Research in context**

**Evidence before this study**

The secretome of irradiated peripheral blood mononuclear cells (PBMCsec) exerts beneficial therapeutic effects in dendritic cell-mediated contact hypersensitivity and lipids found in PBMCsec accounted for the anti-inflammatory actions. Phosphatidylserine (PS), a specific class of lipids, has been shown to inhibit basophil activation, a crucial process during allergies. Whether PBMCsec and PBMCsec-derived lipids are capable of alleviating IgE-dependent allergic reactions has not been investigated so far.

**Added value of this study**

We were able to demonstrate that PBMCsec mitigated compound 48/80 and IgE-induced degranulation of mast cells. Furthermore, we obtained basophils from individuals allergic to birch pollens to show that PBMCsec inhibited allergen-mediated basophil activation. Lipids present in PBMCsec were identified as the active ingredient to prevent immune cell activation.

**Implications of all the available evidence**

The anti-allergic potency of PBMCsec and lipids reported here paves the way for future clinical trials evaluating PBMCsec as a remedy for allergies.

Alt-text: Unlabelled box

## Introduction

IgE-mediated allergies are unwanted, excessive immune responses resulting from an adversely instigated immune system. Worldwide, the prevalence of allergies is on the rise and allergic conditions represent a considerable health concern and socioeconomic burden.^1^^,^^2^ Birch pollen with the major allergen Bet v 1 is a common cause for allergic rhino conjunctivitis and mild asthma in Northern and Central Europe, affecting 8-16 % of the European population.^3^, ^4^, ^5^ Mast cells and basophils are characterized by the expression of the high-affinity receptor for IgE, FcεRI, and, hence, represent the central effector cells of the IgE-mediated allergy.^6^ Both cell types harbour histamine-rich granules which secrete their content upon activation^7^ and are involved in various physiological and pathological conditions, including inflammatory reactions, pathogenic response, immune tolerance, wound healing, and angiogenesis, but also allergy and anaphylaxis.^8^ Basophils are used in basophil activation tests (BAT) where the degree of degranulation following allergen stimulation is determined. This parameter directly correlates with histamine release and, hence, serves as a useful tool to diagnose and monitor allergic disease.^9^

If exposure to the allergen cannot be circumvented, clinical management of allergic symptoms usually involves blocking the action of allergic mediators, e.g. by antihistamines and/or prevention of cell activation and degranulation, e.g. by mast cell stabilizers.^10^ A treatment for allergy with long-term benefit is allergen-specific immunotherapy (AIT) causing immunological changes of the hyper-reactive immune system.^11^ In addition to the modulation of the allergen-specific adaptive immune system, increased activity thresholds of the effector cells mast cells and basophils have been considered to be responsible for allergen-specific unresponsiveness observed following AIT.^11^^,^^12^ Modulating the effector cell reactivity towards allergens therefore represents a promising therapeutic avenue and might help to alleviate the most severe symptoms associated with allergic reactions.

In the early stages of regenerative medicine, stem cells were transplanted to promote post-injury repair. The outcome of these studies were below the expectations, as viability and tissue integration of transplanted cells were poor. More recent reports, however, showed that paracrine factors released by these (dying) cells were sufficient to mediate tissue regeneration observed in studies employing stem cells.^13^^,^^14^ Hence, the therapeutic potential of cell-derived secretomes has become increasingly recognized over time. Interestingly, γ-irradiation of PBMCs was proven to effectively induce cell death and to exert strong tissue-regenerative properties.^15^^,^^16^ Interestingly, irradiation-induced necroptosis was found indispensable for this effect.^17^ Over the past ten years, the secretome of γ-irradiated PBMCs (PBMCsec) has been successfully applied in various indications *in vivo*, including acute myocardial infarction,^18^^,^^19^ chronic ischemic left ventricular dysfunction,^20^ cerebral ischemia,^21^ acute spinal cord injury,^22^ burn injury,^23^ and diabetic wounds.^24^ Furthermore, potent anti-inflammatory actions of PBMCsec have been observed in myocarditis^25^ and contact hypersensitivity.^26^ Functional studies revealed a broad action spectrum of PBMCsec, which covers anti-inflammatory features,^27^^,^^28^ tissue-regenerative,^29^ anti-microbial,^30^ vasodilatory,^31^ and pro-angiogenic^24^^,^^32^ properties. Though strong immunomodulatory effects have been attributed to PBMCsec,^25^ potential anti-inflammatory actions in IgE-mediated allergic reactions have not been elucidated so far. To this end, we sought to determine the effect of PBMCsec on mast cell and basophil activity and to evaluate the anti-allergic potential of PBMCsec.

## Materials and methods

### Generation of PBMCsec

Secretomes of PBMCs were produced in compliance with good manufacturing practice (GMP) by the Austrian Red Cross, Blood Transfusion Service for Upper Austria (Linz, Austria) as described.^24^^,^^26^^,^^33^, ^34^, ^35^ In brief, PBMCs were enriched by Ficoll-Paque PLUS (GE Healthcare, Chicago, IL, USA)-assisted density gradient centrifugation. Cell suspensions were adjusted to a concentration of 2.5 × 10^7^ cells / mL and exposed to 60 Gy γ-irradiation (IBL 437C, Isotopen Diagnostik CIS GmbH, Dreieich, Germany). Cells were cultured in phenol red-free CellGenix GMP DC medium (CellGenix, Freiburg, Germany) for 24 ± 2 hours. Cells and cellular debris were removed by centrifugation and conditioned supernatants containing the secretome were passed through 0.22 µm filters. Viral clearance was performed using Theraflex methylene blue technology (MacoPharma, Mouvaux, France). Secretomes were lyophilized and terminally sterilized by high-dose γ-irradiation (25,000 Gy, Gammatron 1500, Mediscan, Seibersdorf, Austria) as described previously.^22^^,^^34^ CellGenix GMP DC medium used for PBMC culture but without cells served as vehicle control, unless indicated otherwise. The GMP batches A000917399046, A000918399100, and A000918399102 were used in this study.

### Compound 48/80 stimulation and topical application of PBMCsec in mice *in vivo*

For anaesthesia, C57BL/6 mice received intraperitoneal injections of 5 mg/kg body weight xylazin and 100 mg/kg body weight ketamine (both Sigma-Aldrich, St. Louis, MO, USA) and were covered with tissues on a warm plate (30^°^C). Ten µL compound 48/80 (10 mg/mL) were injected intradermally into ear tissues. Twenty-five µL PBMCsec or medium (3:1 in ultrasicc oil-water emulsion base, Hecht-Pharma GmbH, Bremervoerde, Germany) were topically administered 1 hour before and immediately after compound 48/80 injection to obtain a total volume of 50 µL treatment solution. Ear thickness was assessed using an electronic digital micrometre (0-25 mm, Marathon Management Inc, Wilsonville, OR, USA) every 15 minutes up to 90 minutes post injection. N=6 mice receiving anaesthesia only served as controls and n=9 mice were treated with medium or PBMCsec, respectively. Sample sizes were calculated using Piface Application (v1.76, α=0.05, power=0.9)^36^ with 3 means (i.e. levels or treatments), Tukey's HSD method, α=0.05, one-sided, SD=1.22. A sample size of n=25 animals was related to a power>0.9. In total, 24 animals were used in our study, reaching a sufficiently high power of p=0.8894.

### Subcutaneous injection of PBMCsec in mice *in vivo*

*In situ* effect of PBMCsec on dermal mast cells was investigated in a dataset of PBMCsec-treated mice after skin wounding. 14 weeks old, female Balb/c mice were housed under specific-pathogen-free conditions in 12h/12h light/dark cycles and food and water access *ad libidum*. Mice were anesthetized as described above. A 9×9 mm area was excised on shaved backs and left to heal without intervention for 4 weeks. Postoperative analgesia was performed by subcutaneously injecting 0.1 mg/kg body weight buprenorphine (Indivior, North Chesterfield, VA, USA) and 0.125 mg/ml piritramid (Janssen-Cilag Pharma, Vienna, Austria) supplied in drinking water. Four mice were assigned to each treatment condition. Mice received subcutaneous injections of 100 µL PBMCsec (equals to 2.5 U/mL) or medium every other day for 2 weeks. Mice were euthanized on day 14 and 4 mm^2^ biopsies were isolated and subsequently processed for single cell RNA sequencing (scRNAseq).

### Generation of single cell suspensions

Four mm^2^ biopsies of murine skin biopsies were taken and enzymatically digested with MACS Miltenyi Whole Skin Dissociation Kit (Miltenyi Biotec, Bergisch-Gladbach, Germany) for 2.5 hours as recommended by the manufacturer. After dissociation on a GentleMACS OctoDissociator (Miltenyi Biotec), cell suspensions were sequentially passed through 100 µm and 40 µm filters. Viable cells were determined using acridin orange/PI staining and LUNA automated cell counter (both Logos Biosystems, Anyang-si, South Korea). Cell suspensions were adjusted to a concentration of 1 × 10^6^ cells/mL with a viability ≥90 %.

### Generation of single-cell gel-bead in emulsions (GEMs) and library preparation

Immediately after isolation, viable cells were loaded on a 10X chromium controller (10X Genomics, Pleasanton, CA, USA) with a targeted cell recovery of 10,000. For library preparation, Chromium Next GEM Single Cell 3’ Kit v3 was used. cDNA was generated using C1000 Thermal Cycler (Bio-Rad Laboratories, Hercules, CA, USA), GEMs were broken, cDNA was isolated and washed. Libraries were sequenced using the Illumina HiSeq 3000/4000 platform (Illumina, Inc., San Diego, CA, USA) in the 75 bp paired-end configuration. RNA sequencing, demultiplexing, and alignment to a reference genome (mm10) using Cell Ranger Fastq pipeline (10X Genomics) was performed by the Biomedical Sequencing Core Facility of the Centre for Molecular Medicine (CeMM, Vienna, Austria).

### scRNAseq data analysis

Generated scRNASeq data are available in NCBI's Gene Expression Omnibus (GSE202544). For data analysis, Seurat (version 4.0.1, Satija Lab, NYU, NY, USA)^37^^,^^38^ in R (version 3.5.1., The R Foundation, Vienna, Austria) was used. Low-quality cells, empty droplets, cell doublets and multiplets were excluded from analysis before application of the standard workflow for the integration of multiple single cell data sets.^38^ Data were scaled and principal component analysis was performed. Statistically significant principal components were identified by visual inspection. Standard deviation for each principal component was visualized by Elbow plot (Supplemental Figure 1a). Next, we calculated the point where the percent change in variation between consecutive PCs is smaller 0.1% to define the number of PCs to include (Supplemental Figure 1b). Clusters were identified by application of the graph-based clustering approach applied by Seurat. To identify clusters, cluster-specific markers were calculated and gene expressions of cell type-specific established markers were investigated. The ClueGO plug-in^39^ of Cytoscape^40^ was used for functional annotations of differentially expressed genes.

### Isolation and *in vitro* maintenance of primary human dermal mast cells

Mast cell isolation was performed as described previously.^41^ Briefly, abdominal skin and subcutaneous fat tissues were obtained from patients undergoing abdominoplasty. Subcutaneous tissues and reticular dermis were removed surgically. Remaining tissues were minced and subjected to enzymatic digestion (2.4 U/mL dispase II from *Bacillus polymyxa*, Roche, Basel, Switzerland) at 4^°^C overnight. After removal of the epidermis, dermal tissues were digested in collagenase I (Gibco, Thermo Fisher Scientific, Waltham, MA, USA) at 37^°^C for 2 hours. CD117^+^ mast cells were enriched by magnetic cell sorting technology (MACS System, Miltenyi Biotec) as suggested by the manufacturer. To increase purity of isolated cells, the isolation procedure was repeated one more time using the CD117^+^ cell fraction of the first isolation run. Purity was tested by immunofluorescence and was >95 %. CD117^+^ mast cells were cultured in Dulbecco's Modified Eagle Medium (DMEM) supplemented with 10 % (vol/vol) heat-inactivated foetal calf serum (both Gibco), 1 % (vol/vol) penicillin/streptomycin (Biochrom, Berlin, Germany), and 100 ng/mL recombinant human stem cell factor (PeproTech, Rocky Hill, NY, USA). Mast cells were stained with anti-tryptase antibody (#ab2378, RRID: AB_303023, Abcam, Cambridge, UK) according to a published protocol.^41^

### Compound 48/80-induced degranulation of primary human mast cells

Primary human mast cells were seeded at a density of 50,000 to 100,000 per well in flat-bottom 96 well plates in 50 µL DMEM without coloured pH indicator and supplemented with serum and SCF as described above. Cells were pre-treated with 50 µL PBMCsec, 50 µL medium, or 50 µL DMEM (mast cell medium, served as control) overnight. On the following day, cells were washed carefully with 100 µL HEPES (N-2-hydroxyethylpiperazine-N-2-ethane sulfonic acid; 1 M, pH 7.2 to 7.5; Thermo Fisher Scientific) and degranulation was induced by addition of 50 µg/mL compound 48/80 (Sigma Aldrich, (for compound structure see^42^) in 100 µL HEPES. For non-stimulated controls, HEPES without supplements was added. Cells were incubated for 1 hour at 37^°^C with ambient CO_2_. Fifty µL supernatant were separated and preserved and cells (50 µL) were lysed in 150 µL 0.1 % triton X-100 (Sigma Aldrich).

### IgE/anti-IgE-induced degranulation of primary human mast cells

Primary human mast cells were seeded and cultured as described above. Cells were pre-treated with 50 µL PBMCsec, 50 µL medium, or 50 µL DMEM (mast cell medium, served as control) and additionally incubated with 100 ng/mL human IgE (Cat# 401152, RRID: AB_2810964 myeloma, Merck KGaA, Darmstadt, Germany) overnight at 37^°^C with ambient CO_2_. The following day, cells were carefully washed with 100 µL HEPES and degranulation was induced by addition of 5 µg/mL anti-IgE antibody (Cat# 5210-0158, RRID:AB_2773725, Protein Research Products, SeraCare, Milford, MA, USA) in 100 µL HEPES. For non-stimulated controls, HEPES alone was added. Cells were incubated for 1 hour at 37^°^C with ambient CO_2_. Fifty µL supernatant were transferred and preserved and cells (50 µL) were lysed in 150 µL 0.1 % triton X-100 (Sigma Aldrich).

### Beta-hexosaminidase assay

The substrate solution for the beta-hexosiminidase assay was prepared by dissolving 8.9 g disodium-hydrogenphosphate dihydrate (Na_2_HPO_4_·2H_2_O) and 650 mg p-nitro-N-acetyl-beta-D-glucosamide in 400 mL double distilled water and adjusting the pH to 4.5 with 0.4 M citric acid (all reagents Sigma Aldrich). The stop solution consisted of 15.02 g glycine in 900 mL aqua bidest with a pH adjusted to 10.7 using 3 M sodium hydroxide.

Fifty µL substrate solution were added to 50 µL supernatants and 50 µL triton-lysed cells, respectively, and samples were incubated at 37^°^C with ambient CO_2_ for 90 minutes. Seventy-five µL stop buffer were added and optical density was determined at 405 nm using luminometer LUMIstar OPTIMA Reader (BMG LABTECH, Ortenberg, Germany) with FLUOstar OPTIMA software (version 1.20-0, BMG LABTECH).

The percentage of released beta-hexosaminidase was calculated using the following formula^43^:

$$\%=\frac{2\times \text{enzyme}\;\text{in}\;\text{supernatant}}{1\;/\;2\times \;\text{enzyme}\;\text{in}\;\text{supernatant}+4\times \text{enzyme}\;\text{in}\;\text{cell}\;\text{lysate}}\times 100$$

### Compound 48/80 stimulation of human skin biopsies and toluidine blue staining

Abdominal skin was pre-treated with PBMCsec or medium (1:2 dilution) overnight at 37^°^C with 5 % CO_2_. Biopsies were stimulated with 50 µg/mL compound 48/80 for 2 hours at 37^°^C without CO_2_. Samples were formalin-fixed, paraffin-embedded, and cut into 5 µm-thick sections. Slides were de-paraffinised and stained with toluidine blue (0.1% toluidine blue O, Sigma Aldrich, in 1% saline, pH 2.3). Tissues were quickly dehydrated, cleared in xylene and mounted using resinous mounting medium. Sections were analysed on an incident light microscope (BX63, Olympus, Tokyo, Japan) equipped with a 40x objective (UPlanSApo, 40x/0.95, ∞/0.11-0.23/FN26.5) and a UC90 digital camera (both Olympus). Micrographs were acquired using CellSens Dimension software (v2.3, Olympus) with 1/23 seconds exposure time.

### Basophil activation test (BAT)

Generation of recombinant allergens and basophil activation tests were carried out as shown elsewhere.^44^^,^^45^ In brief, heparinized whole blood was obtained from individuals with birch pollen allergy confirmed by case history and allergen-specific IgE levels of >0.35 kU_A_/L in ImmunoCAP (Thermo Fisher Scientific). Different doses of PBMCsec were tested (Supplemental Figure S2) and basophil viability was determined using LIVE/DEAD viability dyes (Thermo Fisher Scientific). Blood was pre-treated with 12.5 U/mL PBMCsec or medium for one hour at 37°C prior to the addition of titrated concentrations of Bet v 1 (1-10 ng/mL), Mal d 1 (10-100 ng/mL), or α-IgE (Cat# 5210-0158, RRID:AB_2773725, 0.5 µg/ml, KPL Protein Research Products, SeraCare, Milford, MA, USA). fMLP (2 µM, Sigma-Aldrich) was used as an IgE-independent stimulus. 0.9% NaCl served as control. Flow cytometric assessment was performed on a BD FACSCanto II flow cytometer using BD FACSDiva software (version 6.1.3) (BD Pharmingen, San Jose, CA, USA). Data were analysed using FlowJo (TreeStar, Inc., Ashland, OR, USA). Basophils were identified by CD123 (peridinin-chlorophyll protein/cyanin 5.5-conjugated mouse anti-human, clone 6H6, Cat# 306002, RRID: AB_314576) and CCR3 (allophycocyanin-conjugated mouse anti-human, clone 5E8, Cat# 310707, RRID: AB_1134156) dual staining (both BioLegend, San Diego, CA, USA) and basophil activation was determined by CD63 expression levels of basophil subpopulations (phycoerythrin-conjugated mouse anti-CD63, clone H5C6, BioLegend, Cat# 353004, RRID: AB_10897809). FcεRIα and phospho-LYN levels of basophil subpopulations were assessed (Alexa Fluor 647-conjugated mouse anti-human FcεRIα, clone AER-37, Cat# 334614, RRID: AB_2168080, BioLegend; rabbit anti-human phospho-LYN, Tyr397, clone E5L3D, #70926, Cell Signalling Technology, Danvers, MA, USA). For blocking experiments, therapeutic anti-human phosphatidylserine antibody (Bavituximab, Creative Biolabs, Shirley, NY, USA) was used. Anti-CD300A neutralizing antibodies was kindly provided by Prof. Dr. Akira Shibuya, Tokyo, Japan.^46^

### Human basophil DNA microarray and data analysis

Whole blood was obtained from individuals allergic to birch pollen and stimulated for 5 hours with PBMCsec or medium. A cohort of 10 Caucasian patients was included in this study. Patient demographics are shown in Table 1. Human basophils were enriched by negative selection using human Basophil Isolation Kit II (Miltenyi Biotec, Bergisch Gladbach, Germany) using autoMACS Pro Separator (Miltenyi Biotec) as recommended by the manufacturer. Negative fractions with a purity of 63% were collected and total RNA was isolated by peqGOLD TriFast (PEGLAB Biotechnologie GmbH, VWR International, Radnor, PA) according to the manufacturer's protocol. RNA quality was determined by Agilent 2100 Bioanalyzer (Agilent Technologies, Santa Clara, CA, USA). Transcriptome profiling was carried out by the Genomics Core Facility at the Medical University of Vienna (Vienna, Austria) using Human Gene 2.1 ST Array (Thermo Fisher Scientific). Transcriptome Analysis Console software (version 4.0, Thermo Fisher Scientific) was used to determine differentially expressed genes by empirical Bayes method and to generate volcano plots. Gene lists of differentially expressed genes (more than 2-fold change of log_2_-tranfsormed expression values) were analysed by Cytoscape (v3.8.5)^40^ using ClueGO (v2.5.7) plug-in.^39^ Biological process, immune system process, molecular function, and KEGG pathways were selected to identify pathways and ontologies. GO terms were visualized by REVIGO software.^47^ Generated basophil sequencing data are available in NCBI´s Gene Expression Omnibus (GSE200490).

**Table 1:** Patient demographics.

Variable	N
Total patients	10
Male	7 (70%)
Female	3 (30%)
Age	
Mean and SEM	37.5 ± 11.2
Median	33
Range	30-60
Ethnicity	
Caucasian	10 (100%)

### Lipid fractionation and lipidomics

Lipid species present in PBMCsec were isolated using a modified Folch method^48^ as described previously.^26^ Lipidomic analysis was performed by reversed-phase high-performance liquid chromatography (HPLC)-electro-spray ionization (ESI)-tandem mass spectrometry (MS/MS).^49^ Samples were screened for phosphatidylserine (PS) 34:0, PS 34:1, PS 36:0, PS 36:1, PS 36:2, PS 36:3, PS 36:4, PS 38:0, PS 38:1, PS 38:2, PS 38:3, PS 38:4, PS 38:5, PS 38:6, PS 40:2, PS 40:3, PS 40:4, PS 40:5, PS 40:6, PS 40:7, PS 40:8, PS 41:4, PS 41:5, PS 41:6, PS 41:7, PS 41:8, PS 42:4, PS 42:5, PS 42:6, PS 42:7, and PS 42:8. Of these species, PS 34:0, PS 36:3, PS 41:6, and PS 41:7 were above the limit of detection.

### Statistical analyses

Data were collected at prospectively defined endpoints and no outliers were excluded. Experiments were repeated using different donors and animals, respectively, in at least triplicates. Confounders were not controlled. Primary cells of one donor were exposed to *in vitro* conditions in parallel. Treatments were not administered blinded. Data were statistically evaluated using GraphPad Prism software (version 5.01, GraphPad Software Inc., La Jolla, CA, USA). Normal distribution of data was determined by graphical means (histograms). Normally distributed data were statistically evaluated by ANOVA and multiple comparison *post hoc* tests with the less conservative Dunnett's or Sidak's correction. Diagrams show arithmetic means and standard errors of the mean (s.e.m.). Box plot diagrams were generated using first and third quartiles as boxes and medians as bars. Whiskers indicate minimum and maximum values, respectively.

### Ethical statements

This study was conducted in accordance with the Declaration of Helsinki and applicable local regulations. Use of human basophils, PBMCs, mast cells, and skin biopsies was approved by the institutional review board of the Medical University of Vienna (Vienna, Austria) (votes 2013/1326, 2017/1539, 1149/2011, and 217/2010). Written informed consent was obtained from all donors.

Animal experiments were performed in accordance with guidelines of the National Institutes of Health guide for the care and use of laboratory animals and approved by the Austrian Federal Ministry of Education, Science, and Research (vote BMBWF-66.009/0075-V/3b/2018).

### Role of funders

Funders had no role in study design, data collection, data analyses, interpretation, or writing of reports.

## Results

### PBMCsec dampens mast cell degranulation-associated tissue swelling *in vivo*

**Figure 1:**
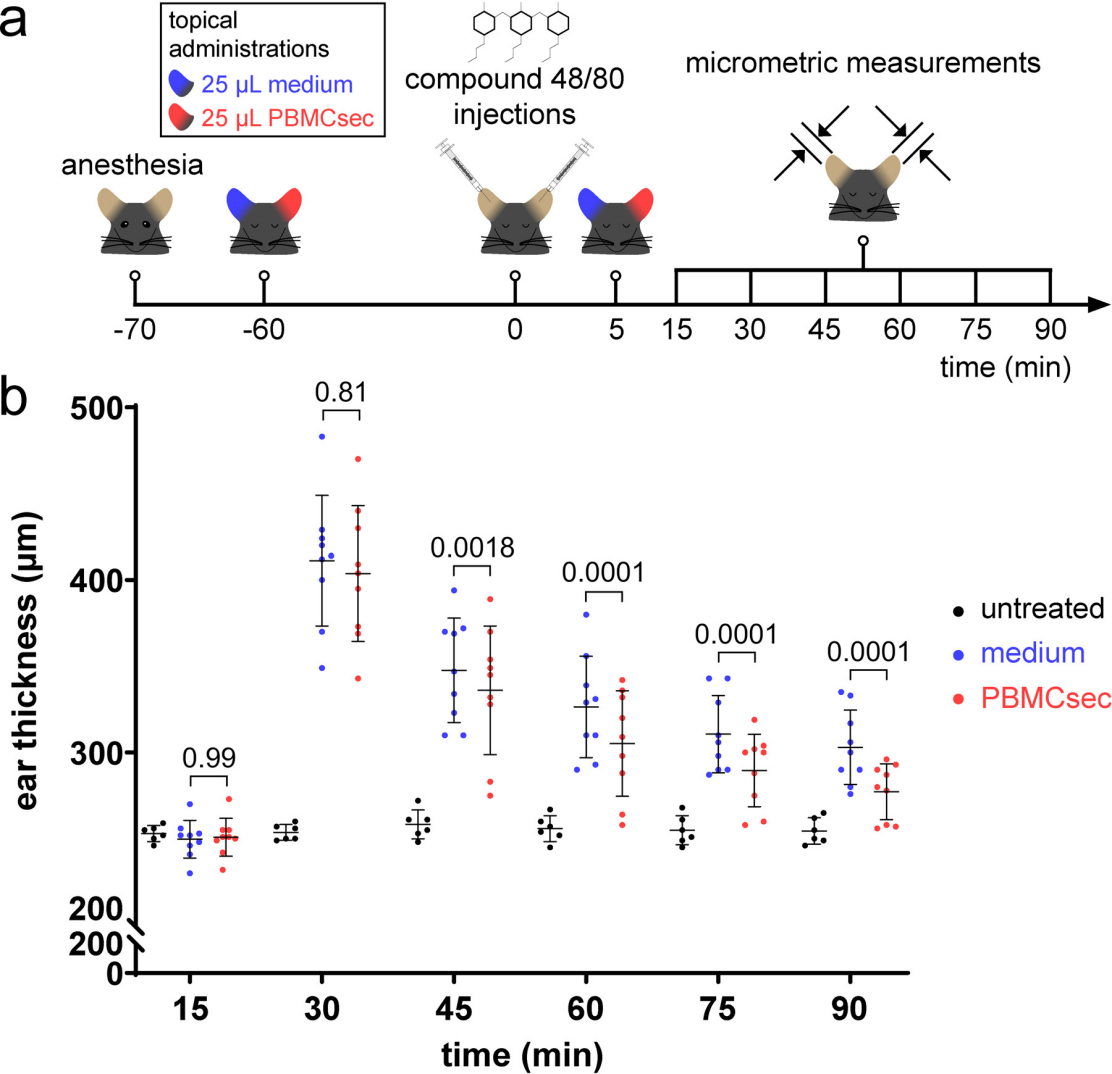
**PBMCsec prevents mast cells activation *in vivo*.** (a) Experimental protocol of topical PBMCsec administration and induction of *in vivo* mast cell degranulation. (b) Ear thickness after intradermal injection of compound 48/80 with topical PBMCsec or medium application. Data show arithmetic means and standard errors of the mean of n=9 mice per treatment condition and n=6 mice for untreated controls. [Treatment groups were compared by two-way, repeated measurements ANOVA and Sidak's multiple comparisons test]. Numbers indicate *p* values of PBMCsec versus medium.

As potent anti-inflammatory effects have already been attributed to PBMCsec,^25^^,^^26^ we sought to determine whether PBMCsec interferes with mast cell activation *in vivo*. Therefore, mice received a single intradermal injection of compound 48/80 after topical pre-treatment with either PBMCsec or medium and ear thickness was tracked over time (Figure 1a). Compound 48/80 induced ear swelling peaking 30 minutes after injection (around 40% increase in ear thickness; Figure 1b), as described previously.^50^ Compared to medium, PBMCsec application dampened compound 48/80-induced ear swelling starting from 30 minutes after injection (351 ± 9.9 µm thickness with medium vs. 332.4± 1.8 µm with PBMCsec, P=0.0018, [ANOVA and Sidak's multiple comparisons test]). These data suggest that topical application of PBMCsec affects mast cell activation and degranulation *in situ*.

### PBMCsec downregulates genes involved in Fc receptor signalling and mast cell degranulation

**Figure 2:**
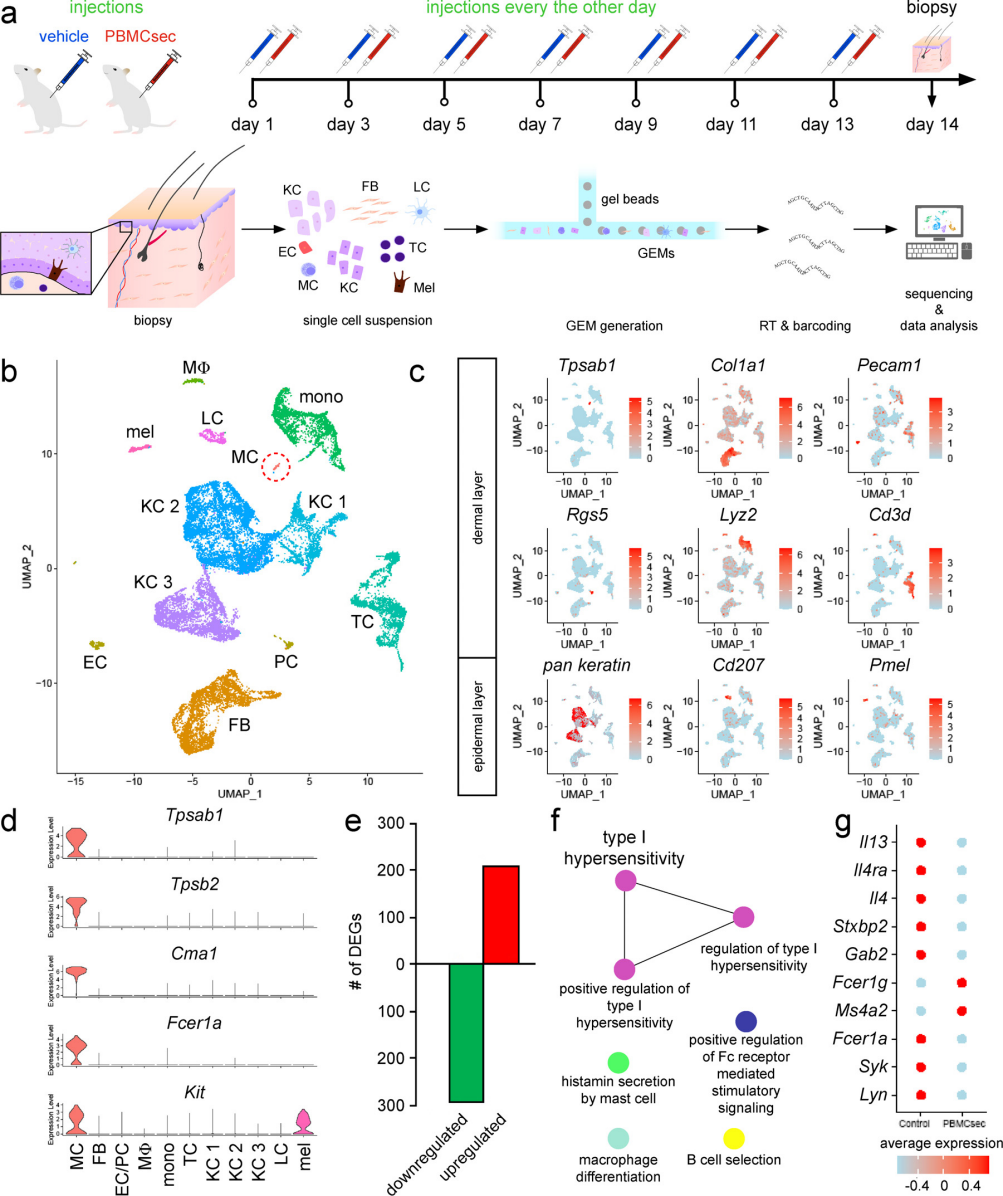
**PBMCsec downregulated Fc receptor signalling and mast cell degranulation in dermal mast cells.** (a) Experimental approach of subcutaneous PBMCsec application *in vivo* and scRNAseq. (b) Uniform manifold approximation and projection (UMAP) plot of PBMCsec- and medium-treated skin. Each dot represents one cell. Colour code indicates identified cell clusters. Dashed circle indicates mast cell population. EC endothelial cells, FB fibroblasts, KC1 keratinocyte cluster 1, KC2 keratinocyte cluster 2, KC3 keratinocyte cluster 3, LC Langerhans cells, MC mast cells, mel melanocytes, mono monocytes, MP macrophages, PC pericytes, TC T cells. (c) Feature plots of biomarker genes used to identify epidermal and dermal cell types. Colour intensities indicate average gene expression levels. Pan keratin feature plot shows blended expressions of keratin 5 (*Krt5*) and *Krt10. Cd3d* Cluster of Differentiation 3d, *Cd207* Cluster of Differentiation 207, *Col1a1* Collagen Type I Alpha 1 Chain, *Lyz2* lysozyme, *Pecam1* Platelet And Endothelial Cell Adhesion Molecule 1, *Pmel* Premelanosome Protein, *Rgs5* Regulator Of G Protein Signaling 5, *Tpsab1* Tryptase alpha/beta 1. (d) Expression of mast cell-specific genes across all cell clusters. *Tpsab1* Tryptase alpha/beta 1, *Tpsb2* Tryptase beta 2, *Cma1* Chymase 1, *Fcer1a* Fc receptor IgE high affinity I alpha polypeptide, *Kit* KIT proto-oncogene receptor tyrosine kinase. (e) Bar diagram showing up- (red) and downregulated genes (green) by PBMCsec compared to medium in dermal mast cells. DEGs were calculated for the mast cell cluster comparing PBMCsec-treated skin versus medium. Genes with average logarithmic fold change of >1.5 or <0.66 were included. (f) GO terms associated with PBMCsec-downregulated genes in mast cells. Each circle represents one GO term. Colour codes indicate biological processes with high amounts of shared genes. (g) Expression of mast cell-specific genes. Colours indicate average gene expressions in medium- and PBMCsec-treated mast cells, respectively. *Il13* interleukin 13, *Il4ra* interleukin 4 receptor alpha, *Il4* interleukin 4, *Stxbp2* syntaxin binding protein 2, *Gab2* growth factor receptor bound protein 2-associated protein 2, *Fcer1g* Fc receptor IgE high affinity I gamma polypeptide, *Ms4a2* membrane-spanning 4-domains subfamily A member 2, *Fcer1a* Fc receptor, IgE, high affinity I, alpha polypeptide, *Syk* spleen tyrosine kinase, *Lyn* LYN proto-oncogene Src family tyrosine kinase.

To investigate the *in vivo* effect of PBMCsec on mast cells in more detail and to identify potential underlying mechanisms, we performed scRNAseq analysis of mice receiving intradermal PBMCsec or medium injections (Figure 2a). All major skin cell types were detected (Figures 2b & c) and a subpopulation of murine dermal mast cells was identified based on high tryptase alpha/beta 1 (*Tpsab1*), tryptase beta 2 (*Tpsb2*), chymase 1 (*Cma1*), Fc receptor, IgE, high affinity I, alpha polypeptide (*Fcer1a*), and KIT proto-oncogene receptor tyrosine kinase (*Kit*) expressions (Figure 2d). Compared to medium, we found that 208 and 292 genes were up- and downregulated by PBMCsec in mast cells, respectively (Figure 2e). Functional annotation of genes downregulated after PBMCsec treatment were associated with a reduction of several immunological processes, such as histamine secretion by mast cells, regulation of Fc receptor-mediated stimulatory signalling pathway, and type I hypersensitivity (Figure 2f). The 208 upregulated genes were associated with intracellular processes, such as electron transport chain, ‘de novo’ protein folding, rRNA binding, and spliceosome (Supplemental Figure S3). We further investigated the cytokine expression levels in dermal mast cells and found that interleukin 4 (*Il4*) and *Il13* were significantly downregulated by PBMCsec compared to medium (Figure 2g). Furthermore, expressions of Il4 receptor alpha (*Il4ra*), syntaxin binding protein 2 (*Stxbp2*), and growth factor receptor bound protein 2-associated protein 2 (*Gab2*) were diminished in PBMCsec-treated mast cells (Figure 2g). While *Fcer1a* was significantly downregulated in mast cells after topical administration of PBMCsec, expression of other subunits of the Fcε receptor (*Fcer1b* and *Ms4a2*) was induced. Interestingly, expressions of *Lyn* and *Syk*, two tyrosine kinase involved in Fcε receptor signalling, were significantly inhibited by PBMCsec. These data indicate that PBMCsec prevents mast cell degranulation, at least partially, by transcriptional downregulation of mast cell-specific genes.

### PBMCsec abrogates degranulation of human primary skin mast cells

**Figure 3:**
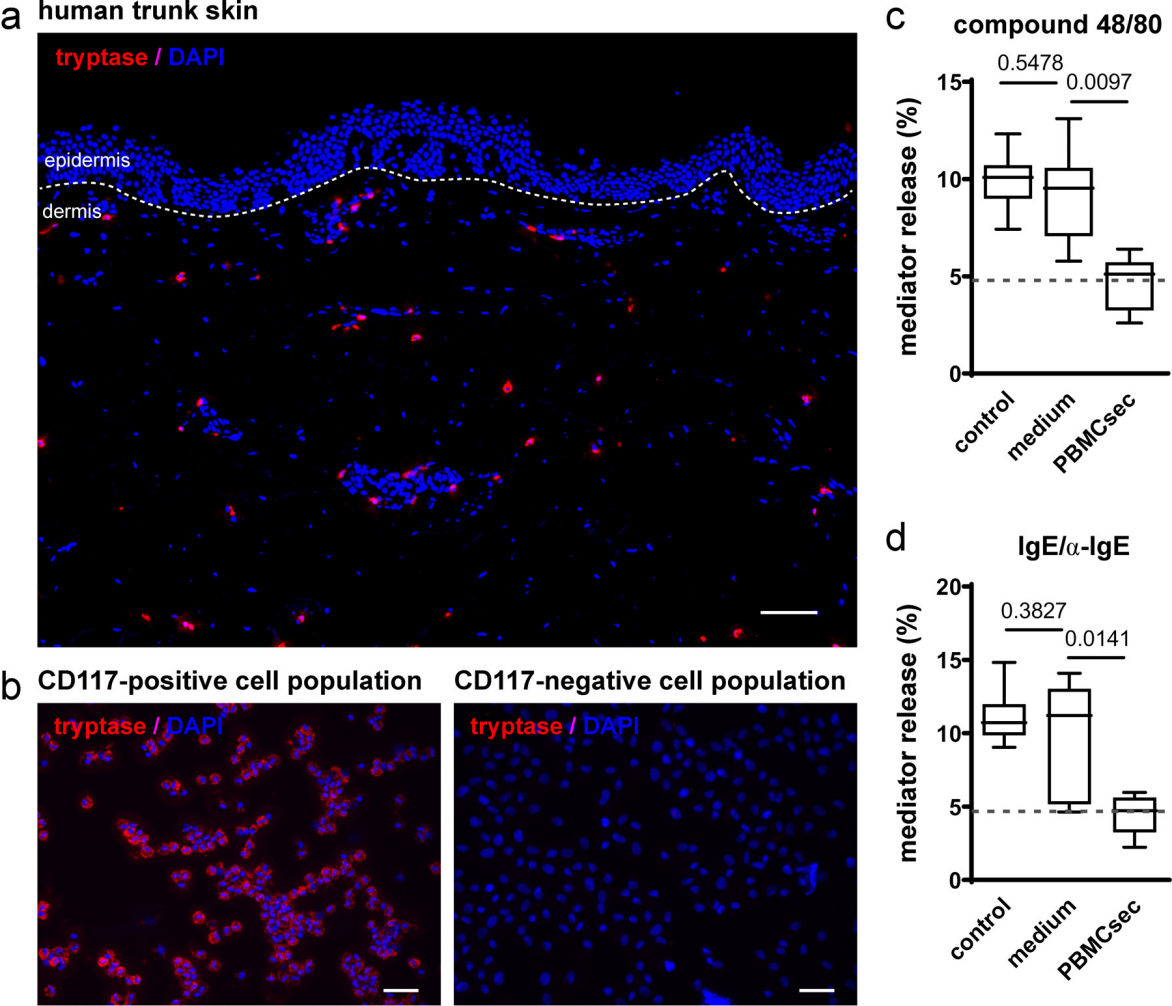
**PBMCsec prevents compound 48/80- and IgE/α-IgE-induced mediator release by human dermal mast cells.** (a) Human dermal mast cells, indicated by tryptase-positive staining, were isolated from trunk skin and (b) enriched by CD117 cell sorting. Scale bar in (a) 100 µm. Scale bars in (b) 50 µm. Mast cell degranulation was assessed by β-hexaminidase release upon (c) compound 48/80 and (d) IgE/α-IgE stimulation of primary human dermal mast cells. Controls refer to medium routinely used to culture mast cells (DMEM alone), while medium represents the medium used for PBMC culture and secretome generation. Dashed horizontal lines in (c) and (d) indicate levels of mediator release of non-stimulated mast cells. Data show averages of n=4 donors from independent experiments. [P values were computed using one-way ANOVA and Dunnett's multiple comparisons tests].

After studying the effect of PBMCsec on murine mast cells *in vivo*, we investigated whether human mast cells were similarly affected by PBMCsec. Since human mast cell lines often fail to accurately reflect the behaviour of primary mast cells, we purified primary human mast cells from abdominal skin using CD117^+^ magnetic beads (Figures 3a & b). Mast cells were treated with PBMCsec or medium and β-hexosaminidase release, an indicator of mast cell degranulation, was assessed after stimulation with compound 48/80. Mast cell isolation and subsequent *in vitro* culture displayed little effect on mediator release (Figures 3c & d). While mediator release was comparably induced by compound 48/80 in control (10.0 ± 1.4%) and medium (9.1±2.4%, P*=*0.5478 versus control, [one-way ANOVA and Dunnett's multiple comparisons test]) mast cells, PBMCsec pre-treatment largely attenuated β-hexosaminidase secretion after stimulation with compound 48/80 (4.6±1.4%; Figure 3c, P=0.0097 versus medium, [one-way ANOVA and Dunnett's multiple comparisons test]). Since compound 48/80 is a synthetic substance with limited physiological relevance, we next investigated whether IgE/α-IgE stimulation was also counteracted by PBMCsec. Addition of IgE/α-IgE led to increased release of β-hexosaminidase, comparable to that observed with compound 48/80 (11.1±1.8% in controls and 9.7±3.9% with medium after IgE/α-IgE stimulation; Figure 3d, P=0.3827, [one-way ANOVA and Dunnett's multiple comparisons test]). In contrast, pre-treatment with PBMCsec remarkably prevented IgE/α-IgE-induced mediator release compared to medium (4.4±1.3%, P=0.0141, [one-way ANOVA and Dunnett's multiple comparisons test]). We further assessed the effect of PBMCsec on mast cell degranulation *in situ* and observed that PBMCsec prevented compound 48/80 treatment-induced degranulation of skin-resident mast cells (Supplemental Figure S4). Together these findings indicate that PBMCsec is able to interfere with compound 48/80-induced and IgE/α-IgE-induced mast cell degranulation in the skin.

### PBMCsec impairs allergen-dependent activation of basophils

**Figure 4:**
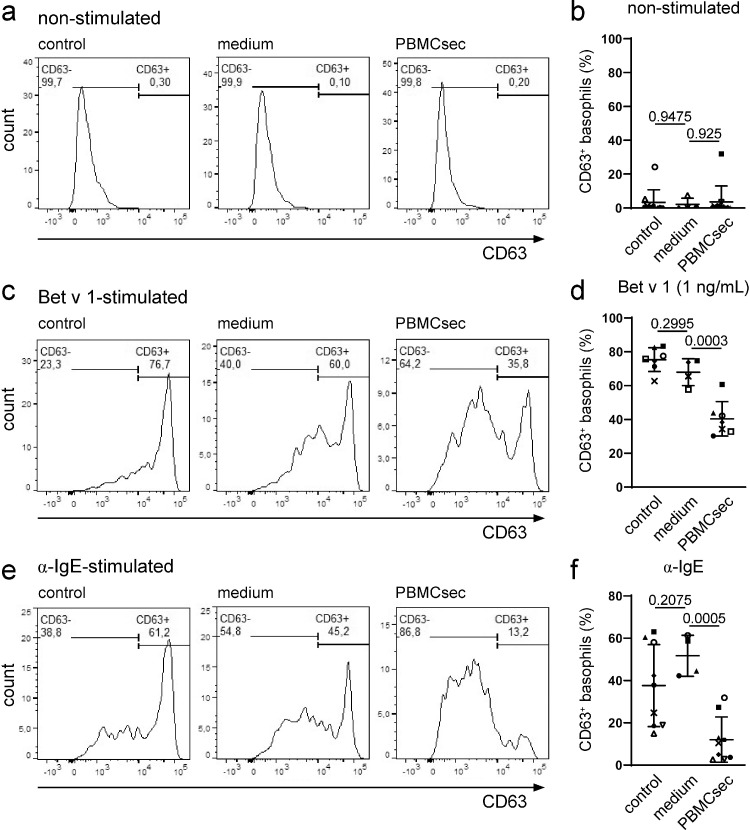
**PBMCsec prevents allergen-mediated basophil activation.** Representative histograms of (a) non-stimulated, (c) Bet v 1-, and (e) α-IgE-induced basophil activation after gating on lymphocytes and monocytes in FSC/SSC and CD123^+^, CCR3^+^ cells. Statistical analyses of (b) non-stimulated, (d) Bet v 1-, and (f) α-IgE-induced activation of PBMCsec- or medium-treated human basophils. Controls refer to cells treated with 0.9 % NaCl. Each symbol identifies one individual donor. Horizontal lines indicate arithmetic means and standard deviations, respectively. [Data were compared by ordinary one-way ANOVA and Dunnett's multiple comparisons *post hoc* analysis].

The encouraging findings obtained from mast cells prompted us to investigate whether PBMCsec exerted a similar effect on allergen-activated primary human basophils. Therefore, the anti-allergic potential of PBMCsec was assessed in basophil activation tests with Bet v 1 and cells from birch pollen-allergic individuals to perform allergen-specific effector cell activation. Pre-incubation of PBMCsec and medium had no effect on spontaneous degranulation (Figures 4a & b) and viability of PBMCsec-treated basophils was above 97 % (Supplemental Figure S5). Addition of Bet v 1 strongly induced surface expression of the basophil activation marker CD63 (75.4±7.0% CD63^+^ cells in controls; Figures 4c & d, Supplemental Figure S6). While medium showed no effect on basophil activation (68.0±8.0% CD63^+^ cells, P=0.2995 versus controls, [one-way ANOVA and Dunnett's multiple comparisons test]), PBMCsec strongly inhibited basophil activation with Bet v 1 (40.4±10.25% CD63^+^ cells; Figures 4c & d, P=0.0003 versus medium, [one-way ANOVA and Dunnett's multiple comparisons test]). Since IgE antibodies from individuals with birch pollen allergy are known to cross-react with the Bet v 1-homologous major apple allergen Mal d 1, we used this allergen as a second stimulus. Basophil activation after exposure to 10 ng/mL Mal d 1 (57.9±21.4% activated basophils in controls, 51.1±22.2% CD63^+^ cells with medium, P=0.852 controls versus medium, [one-way ANOVA and Dunnett's multiple comparisons test]; 26.1±17.5% CD63^+^ cells with PBMCsec, P=0.1992 versus medium, [one-way ANOVA and Dunnett's multiple comparisons test]; Supplemental Figure S7) and 100 ng/mL Mal d 1 (33.9±16.2% activated basophils in controls, 27.2±14.8% CD63^+^ cells with medium, P=0.7075 controls versus medium, [one-way ANOVA and Dunnett's multiple comparisons test]; 8.4±6.7% CD63^+^ cells with PBMCsec, P=0.1298 versus medium, [one-way ANOVA and Dunnett's multiple comparisons test]; Supplemental Figure S8) was strongly diminished by PBMCsec. We next investigated whether PBMCsec was also able to inhibit basophil degranulation after stimulation with α-IgE. While controls and medium-treated basophils displayed high activation levels after addition of α-IgE (37.6±19.4% activated basophils in controls and 51.7±9.7% CD63^+^ cells with medium treatment*,* P=0.2075, [one-way ANOVA and Dunnett's multiple comparisons test]), PBMCsec largely abrogated α-IgE-induced basophil activation (12.0±10.7% CD63^+^ cells, P=0.0005 versus medium, [one-way ANOVA and Dunnett's multiple comparisons test]; Figures 4e & f). Interestingly, we observed only diminished inhibitory activity of PBMCsec when basophils were exposed to fMLP, an IgE- and FcεrI-independent stimulus (47.2±20.6% activated basophils with medium and 33.9±21.2% CD63-positive basophils with PBMCsec, P=0.3069 versus medium, [one-way ANOVA and Dunnett's multiple comparisons test]; Supplemental Figure S9). These data demonstrate that PBMCsec impairs IgE-dependent basophil activation.

### PBMCsec curbs Fc-receptor signalling and immune cell degranulation pathways in basophils

**Figure 5:**
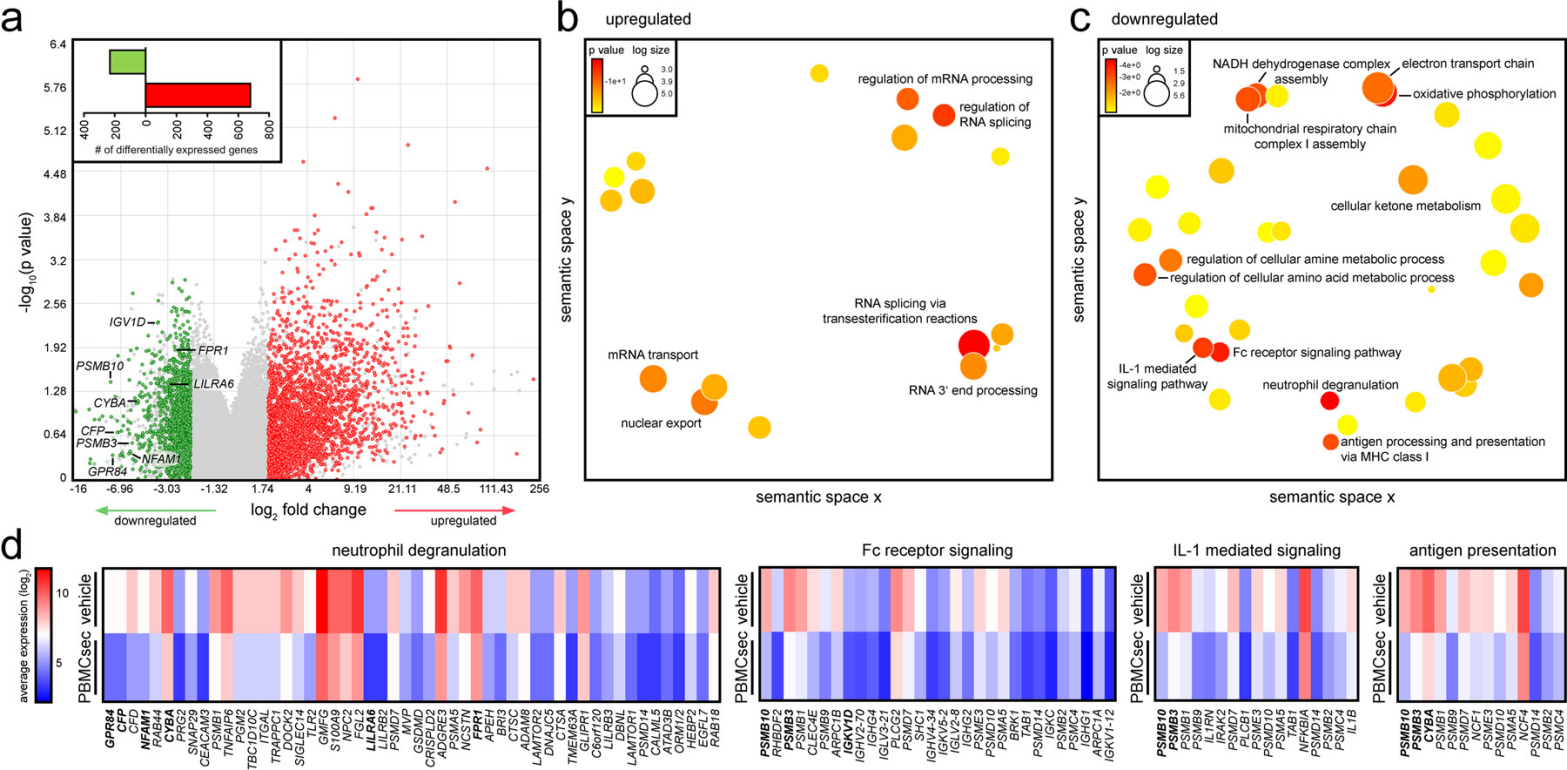
**PBMCsec downregulates mast cell degranulation-associated genes in individuals allergic to birch pollens.** (a) Volcano plot of down- (green) and up- (red) regulated genes by PBMCsec compared to medium. Each dot represents one gene. Cut-offs of average <0.5 and >2-fold change were set. Bar diagram indicates absolute numbers of differentially expressed genes by PBMCsec compared to medium. Gene ontologies associated with (b) up- and (c) down-regulated genes by PBMCsec compared to medium. Each circle represents one gene ontology. Colours indicate *p*-values. (d) Downregulated genes associated with ontologies. Colour code indicates gene expression levels of medium- and PBMCsec-treated human basophils.

To elucidate potential underlying mechanisms by which PBMCsec prevents allergen-dependent effector cell activation, we performed transcriptomic analyses of PBMCsec- and medium-treated basophils obtained from allergic individuals. We detected a total of 909 differentially expressed genes, whereby 231 genes were down- and 678 genes were upregulated by PBMCsec compared to medium, respectively (Figure 5a, insert). Gene ontology enrichment analyses revealed that upregulated genes were implicated in RNA processing and splicing (Figure 5b), while downregulated genes were mainly associated with immunological processes, including neutrophil degranulation, Fc-receptor signalling, IL-1 mediated signalling, and antigen processing and presentation (Figure 5c), corroborating our data obtained from PBMCsec-treated murine dermal mast cells. Downregulated genes associated with neutrophil degranulation included *GPR84, NFAM1, CYBA*, and *LILRA6* (Figure 5d, Supplemental Figure S10). Several genes encoding proteasomal subunits were downregulated by PBMCsec, such as *PSMB3* and *PSMB10*, which are involved in Fc-receptor signalling, IL-1 signalling, as well as antigen processing and presentation (Figure 5d, Supplemental Figure S10). Since transcriptomic data pointed towards diminished expression of FcεR signalling- and degranulation-associated genes, we further investigated these pathways on protein level. FcεRI expression remained unaffected after 1 hour PBMCsec treatment with either Bet v 1 or α-IgE challenge compared to medium (Supplemental Figure S11). We also observed no effect of PBMCsec on FcεRI levels after 24 hours incubation (Supplemental Figure S12). Intriguingly, phosphorylation levels of LYN were not altered by PBMCsec in response to Bet v 1 stimulation (Supplemental Figure S13). We further investigated the expression patterns of inhibitory receptors expressed by basophils. While *CD300A* mRNA and CD300A surface expression were found upregulated in non-activated PBMCsec-treated basophils compared to medium (Supplemental Figure S14), histamine receptor H2 (*HRH2*), sialic acid binding Ig like lectin 7 (*SIGLEC7*), and *SIGLEC8* were found decreased in PBMCsec compared to medium (Supplemental Figure S15). These data suggest that PBMCsec prevents basophil activation presumably by inhibiting downstream Fc receptor signalling.

### Lipids present in PBMCsec inhibit mast cell degranulation and basophil activation

**Figure 6:**
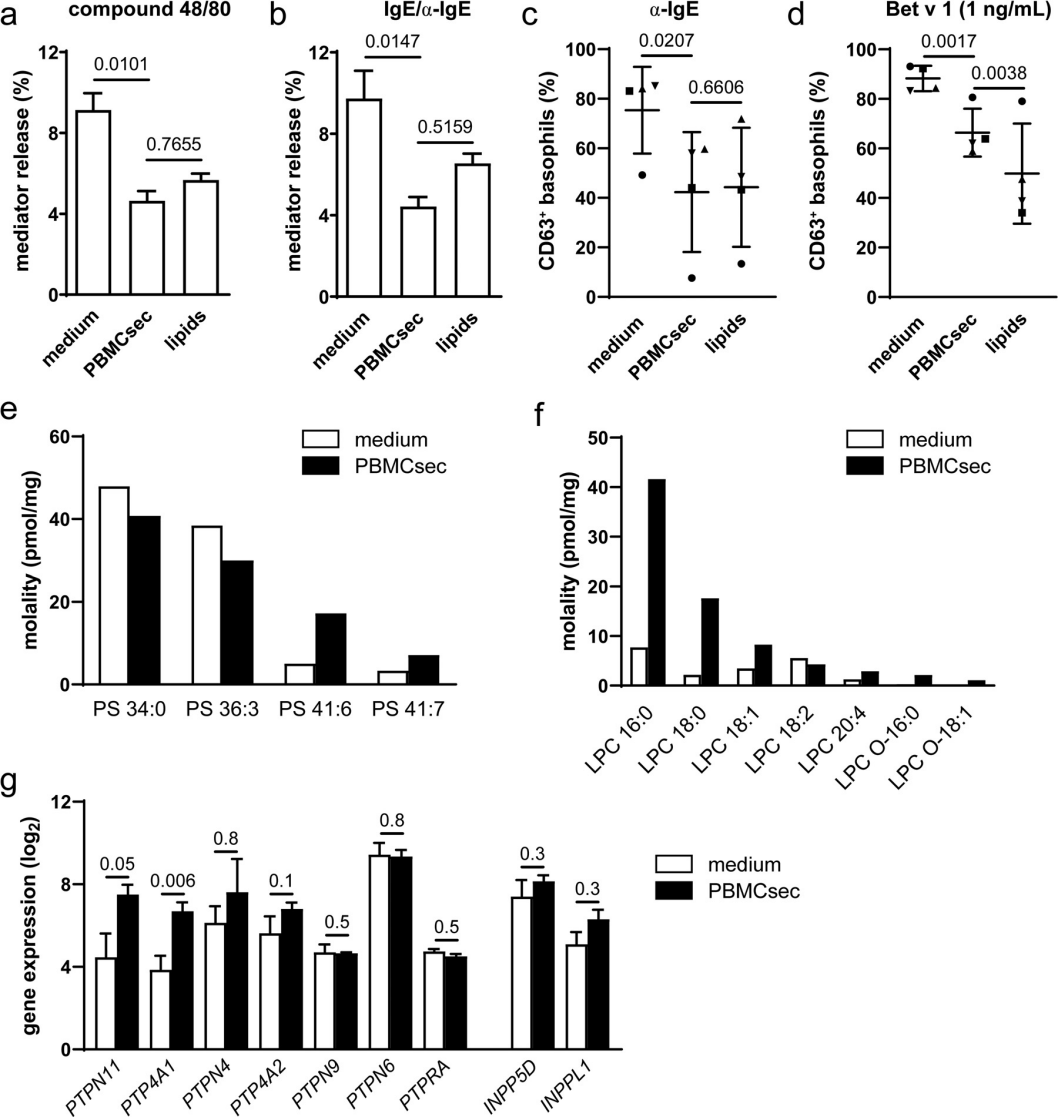
**Lipids prevent mast cell degranulation and basophil activation.** Mast cells were pre-treated with medium, PBMCsec, or PBMCsec-derived lipids and mediator release was assessed following (a) compound 48/80 and (b) IgE/α-IgE stimulation. Bars show mean ± s.e.m. of n=4 donors for medium and PBMCsec and n=2 for lipids. Data were compared by ANOVA and Sidak's multiple comparisons test. Basophil activation test after pre-incubation with PBMCsec-derived lipids upon (c) α-IgE and (d) Bet v 1 (1 ng/mL) stimulation. Each dot represents one donor. [Data were compared by ANOVA and Dunnett's multiple comparisons test]. (e) PS and (f) LPC lipid species in PBMCsec and medium quantified by lipidomics. LPC lysophosphatidylcholine, PS phosphatidylserine. (g) Gene expression of non-receptor protein tyrosine and lipid phosphatases in basophils obtained from individuals allergic to birch pollen. Data of n=3 individual donors were compared by empirical Bayes method and are presented as mean±s.e.m. *INPPL1* inositol polyphosphate phosphatase-like 1, *INPP5D* inositol polyphosphate-5-phosphatase D, *PTPN* protein tyrosine phosphatase non-receptor type, *PTP4A1* protein tyrosine phosphatase type IVA member 1, *PTP4A2* protein tyrosine phosphatase type IVA member 2, *PTPRA* protein tyrosine phosphatase receptor type A.

Finally, we sought to identify the molecular class of active ingredients that mediates the anti-allergic action of PBMCsec. As a therapeutic effect of PBMCsec-derived lipids in delayed type hypersensitivity has already been demonstrated,^26^ we questioned whether lipids might also be capable of preventing effector cell activity in IgE-mediated allergy. To this end, we treated human mast cells with lipids isolated from PBMCsec prior to stimulation with compound 48/80 or IgE/α-IgE. Compound 48/80-induced mast cell degranulation was diminished both by PBMCsec (4.6±1.4% released mediator) and lipids (5.7±0.5% mediator release) compared to medium (9.1±2.4%, P=0.0101 PBMCsec versus medium and P=0.7655 PBMCsec versus lipids, [ANOVA and Sidak's multiple comparisons test]; Figure 6a). Comparably, mediator release was abolished by PBMCsec (4.4±1.3% β-hexosaminidase release) and lipids (6.5±0.7%) following IgE/α-IgE stimulation (medium: 9.7±3.9%, P=0.0147 PBMCsec versus medium and P=0.5159 PBMCsec versus lipids, [ANOVA and Sidak's multiple comparisons test]; Figure 6b). Similarly, lipid pre-treatment prevented basophil activation following α-IgE to a similar extent as PBMCsec (P=0.0207 PBMCsec versus medium and P=0.6606 PBMCsec versus lipids, [ANOVA and Dunnett's multiple comparisons test]; Figure 6c) and even stronger than PBMCsec after stimulation with Bet v 1 (P=0.0017 PBMCsec versus medium and P=0.0038 PBMCsec versus lipids, [ANOVA and Dunnett's multiple comparisons test]; Figure 6d, Supplemental Figure S16). Previously, an inhibitory effect of phosphatidylserine (PS) on basophils via CD300a has been reported.^46^^,^^51^ Since γ-irradiated PBMCs, the source of PBMCsec, express PS on their surface,^17^^,^^52^ we screened PBMCsec for the presence of PS analytes by lipidomics. Among all PS species analysed, we were able to detect PS 34:0, PS 36:3, PS 41:6, and PS 41:7 in our samples. Interestingly, PS 34:0 and PS 36:3 were the most abundant in medium, while levels of PS 41:6 and PS 41:7 were higher in PBMCsec compared to medium (Figure 6e). Since we observe no inhibitory effect of medium on mast cell degranulation (Figures 3c & d), these data suggest that specifically PS 41:6 and PS 41:7 enriched in PBMCsec might account for the inhibitory effect of PBMCsec-derived lipids on mast cells. To determine whether PBMCsec- and, more specifically, lipid-mediated inhibition of basophils occurs in an PS-CD300A-dependent manner, this pathway was blocked by Annexin V and antibodies targeting PS and CD300A, respectively. Neutralizing components of the PS-CD300A signalling axis showed no effect on lipid-mediated prevention of basophil activation (Supplemental Figures S17-S19). We thus extended our analysis to further lipid species and detected several lysophosphatidylcholines (LPC) (Figure 6f), lysophosphatidylethanolamines (LPE), phosphatidylcholines (PC), and phosphatdiylethanolamines (PE) (Supplemental Figure S20).

To further characterize the lipid-mediated inhibitory effects, we investigated genes implicated in lipid mediator synthesis and metabolism in allergic basophil donors. We detected no effect of PBMCsec on the expression of arachidonate lipoxygenases, cytochrome c oxidases, or leukotriene- and prostaglandin-associated genes except for *PTGER4* (Supplemental Table S1). We then proceeded to analyse non-receptor protein tyrosine and lipid phosphatases and observed a PBMCsec-induced upregulation of protein tyrosine phosphatase non-receptor type 11 (*PTPN11*) and protein tyrosine phosphatase 4A1 (*PTP4A1*), genes coding for Src homology 2 domain-containing protein tyrosinephosphatase 2 (SHP-2) and phosphatase of regenerating liver-1 (PRL1), respectively (Figure 6g).

## Discussion

The activation of mast cells and basophils by allergens represents a crucial immunological event in allergic reactions as these effector cells are the major source of histamine, and thus, key players in the pathophysiology of IgE-mediated allergy. Both cell types originate from a common CD34^+^ progenitor and respond to IgE receptor crosslinking by vast release of mediators, lipid species, cytokines, chemokines, and proteases. The exact composition of the secreted factors and the responses to certain stimuli, however, are highly cell type-specific. While histamine, granzyme B, and VEGFs are released by both cell types, IL-4 is predominantly secreted by basophils and tryptase by mast cells. Thus, the functional heterogeneity of these cells has often hampered development of effective anti-allergic pharmacological agents acting on both cell types simultaneously.^53^ In the current study, we provide evidence that the secretome of γ-irradiated PBMCs has the ability to inhibit both cell types.

Currently used allergy remedies include antihistamines which antagonize the actions of histamine by selectively blocking histamine H_1_ receptor.^10^ As PBMCsec prevented IgE-induced mast cell degranulation and mediator release, blocking the action of released histamine seems as an unlikely mode of action. Mast cell stabilizers are often used to treat allergies, especially when antihistamines are not well tolerated. Their modes of action include inhibition of signal transduction or prevention of calcium influx and release.^54^^,^^55^ Nonetheless, the exact therapeutic action of several mast cell stabilizers remains elusive and targeting additional inflammatory events might complement their effect on mast cell stabilization.^55^ Future studies will be needed to investigate whether PBMCsec belongs to the class of mast cell stabilizers and whether it interferes with intracellular calcium signalling. In addition to antihistamines and mast cell stabilizers, decongestants are widely used to target the adrenergic receptor system, thereby causing vasoconstriction. Since PBMCsec is reportedly vasodilatory,^31^ it remains to be determined whether secretome-induced vasodilatation might represent a possible side effect and whether a combinational therapy of PBMCsec and decongestants might be advised for allergy treatment. As PBMCsec has already demonstrated potent anti-inflammatory properties in various inflammatory diseases and is capable of modulating the activity of several cell types,^25^^,^^26^ PBMCsec might help alleviating allergic symptoms not solely by directly inhibiting mast cell degranulation and basophil activation, but in a more pleiotropic manner and future studies are required to elucidate the full intricate picture of secretome-mediated allergy relief.

Mechanistically, our data suggest blockade of FcεR signalling by PBMCsec, thereby inhibiting activation and degranulation of mast cells and basophils. Although PBMCsec contains proteases,^18^ surface expression of FcεR1 was not affected by PBMCsec treatment and phosphorylation of LYN, an FcεR1 down-stream tyrosine kinase involved in basophil activation,^56^ remained unaltered by PBMCsec. Thus, PBMCsec inhibits basophil activation downstream of FcεRI and LYN at yet unknown points of the signalling cascades engaged by IgE and allergens. However, our data suggest several plausible mechanisms. Stxbp2 is involved in intracellular trafficking and vesicle fusion with membranes. Reduced *Stxbp2* expression has been shown to impair mast cell degranulation^57^ and we observed diminished *Stxbp2* expression in mast cells after stimulation with PBMCsec. Moreover, Gab2 plays an essential role in FcεR-mediated signalling^58^ and *Gab2* expression was reduced in PBMCsec-treated mast cells. Hence, targeting the vesicle trafficking machinery and/or downregulation of *Gab2* might represent conceivable mechanisms by which PBMCsec inhibits FcεR signalling and immune cell activation in the allergy context. IL-4 and IL-13 secretion is a characteristic feature of mast cells and PBMCsec diminished cytokine expressions and cytokine responses in dermal mast cells. Inhibition of immune cell-specific cytokine secretion patterns by PBMCsec has already been demonstrated in dendritic cells^26^ and macrophages.^28^ Modulating immune cell activities, especially the secretory behaviour, thus represents an additional mode of action of PBMCsec.

Inhibitory surface receptors are therapeutically attractive targets. The inhibitory histamine receptor H2R was upregulated during allergen-specific immunotherapy and was responsible for basophil inhibition.^59^ Siglec-7^60^ and Siglec-8^61^^,^^62^ have further been reported as key inhibitory receptors on mast cells and basophils. In our data, we found that *HRH2, SIGLEC7*, and *SIGLEC8* were strongly reduced by PBMCsec and these molecules therefore do not represent the factors by which PBMCsec mediates basophil inhibition. Downregulation of the inhibitory receptors might serve as a compensatory mechanism in response to impaired basophil activation by PBMCsec. Intriguingly, *CD300A* was the only inhibitory surface receptor induced by PBMCsec. An inhibitory effect of CD300a has been demonstrated previously by Sabato and colleagues who showed that apoptotic cells prevented Bet v 1-induced CD63 upregulation in basophils of individuals allergic to birch pollens.^51^ Mechanistically, the authors reported that binding of the apoptosis-related signal molecule phosphatidylserine (PS) to CD300a inhibited IgE/FcεRI-dependent basophil degranulation.^46^^,^^51^ Previously, we successfully employed PBMCsec in a mouse model for DNFB-induced contact hypersensitivity and identified the lipid fraction as the major active ingredients to mediate the pharmacological effect.^26^ These findings are in line with results of the current study, where we provide evidence that PBMCsec-derived lipids were predominantly accountable for the inhibitory effect of PBMCsec on mast cells and basophils. Furthermore, we were able to confirm the presence of a distinct subset of PS species in PBMCsec and showed that *CD300A* was the only inhibitory surface receptor in human basophils induced by PBMCsec. Intriguingly, CD300a recruits SHP-1 and SHP-2 upon PS stimulus^63^, ^64^, ^65^ and we observe a strong induction of the SHP-2 gene *PTPN11* by PBMCsec which reportedly exerts inhibitory effects.^66^, ^67^, ^68^, ^69^ Nonetheless, blockade of the PS-CD300A axis did not abrogate the PBMCsec- and lipid-mediated inhibition of basophil activation, suggesting that either PS species are not involved in the regulation of mast cell and basophil degranulation, or PS represent just one of several lipid species showing comparable effects and blocking of PS alone is therefore not sufficient to inhibit the overall lipid activity. For instance, we detected several lysophosphatidylcholines (LPC) in the lipid fraction of PBMCsec and LPC have recently been shown to inhibit human eosinophil activation.^70^ However, as blocking antibodies are not available for most of the identified lipid classes, deciphering the lipid(s) responsible for the observed effect is difficult and requires more sophisticated experiments.

In spite of all efforts, our study is subject to some limitations. The fact that the lipid species responsible for the observed effect is still not known represents a significant limitation of this study. Furthermore, the rather low purity of isolated basophils after negative selection might impact bioinformatics analyses. Some of the identified genes are not exclusively expressed in basophils, but are rather also implicated in similar pathways in other granulocyte populations, mainly neutrophils. This could impact the downstream gene ontology analysis. In our study, 10 volunteer (70% male and 30% female) individuals allergic to birch pollens were used to study the effect of PBMCsec on basophil activation. As allergies are known to display a high variability between different phenotypes, more pre-clinical studies with higher sample size numbers are warranted to investigate individual forms and manifestations of the disease prior to first-in-men studies. Atopic dermatitis, for instance, displays different disease phenotypes depending on age, chronicity, IgE status, but also ethnicity, amongst other factors.^71^, ^72^, ^73^ Here we only investigated patients of Caucasian ethnicity. As ethnicity represents an important aspect for the development of targeted and personalized therapies,^71^^,^^72^ it remains to be determined whether the beneficial effects we were able to detect in our cohort can likewise be observed in other ethnic groups. In addition, confounders were not controlled in our study. Nonetheless, the IgE status of all participating individuals was determined prior to inclusion in our study.

In pre-clinical toxicological tests, PBMCsec has been successfully employed without causing major adverse events after topical^74^ and intravenous application (study number 35015, Ankersmit HJA, unpublished). Efficacy of viral clearance was demonstrated^34^ and safety and tolerability of topically administered autologous PBMCsec has already been reported (clinicaltrials.gov identifier NCT02284360).^75^ Allogeneic PBMCsec in a gel formulation for topical application is currently being tested in a first-in-men phase I/II study for treatment of diabetic foot ulcers (EudraCT number 2018-001653-27, clinicaltrials.gov identifier NCT04277598).^35^ The therapeutically most effective mode and time point(s) of administration for allergic conditions are yet to be determined. Topical application of water-oil emulsions, nasal sprays, eye drops, nebulization solutions, injections, or oral administration are conceivable routes of administration, depending on the tissues most affected by allergic symptoms. The current study together with our previous work pave the way for future (pre-)clinical studies to evaluate the therapeutic efficacy of PBMCsec to alleviate allergic symptoms.

## Contributors

ML, GSA, BB, HJA, and MM: design of the study. ML, GSA, CK, DC, LMA, VV, and AP: data acquisition. ML, GSA, CK, DC, FG, LMA, VV, AG, MD, KK, DB, BB, HJA, and MM: data analyses and interpretations. AS, BB, HJA, MM: resources. ML, FG, LMA, MM: access to the data and data verification. ML, BB, MM, and HJA: drafting original manuscript. All authors have review and approval of the final manuscript.

## Declaration of interests

The Medical University of Vienna has claimed financial interest. HJA holds patents related to this work (WO2010079086A1; WO2010070105A1; EP3502692A1; WO2021130305A1). MM hold a patent related to this work (WO2021130305A1). ML, DC, VV, AG, MD, KK, DB, AP, and HJA are affiliated with the company Aposcience AG, a manufacturer of PBMCsec. All other authors declare no potential conflicts of interest.

## Data sharing statement

Generated scRNASeq data (GSE202544) and basophil sequencing data (GSE200490) are available in NCBI´s Gene Expression Omnibus (GEO).

## Appendix Supplementary materials

Image, application 1

Image, image 2

Image, image 3

Image, image 4

Image, image 5

Image, image 6

Image, image 7

Image, image 8

Image, image 9

Image, image 10

Image, image 11

Image, image 12

Image, image 13

Image, image 14

Image, image 15

Image, image 16

Image, image 17

Image, image 18

Image, image 19

Image, image 20

Image, image 21

Image, application 2
