# Supplementary material for: The secretome of irradiated peripheral blood mononuclear cells attenuates activation of mast cells and basophils
Source: eBioMedicine. 2022 Jun 4;81:104093. doi: 10.1016/j.ebiom.2022.104093 (PMC9168057; doi:10.1016/j.ebiom.2022.104093)
Supplement: Supplementary file 1 [file mmc1.docx]

# **Supplemental Figure S1. Elbow plot for identification of principal components to include.** (a) Elbow plot showing the standard deviation for every principal component. Number of dimension = 50. (b) PCs with variation greater than 0.1% are shown in red while PCs with variation below 0.1%. are illustrated in lighblue. According to the analysis 21 PCs were included for downstream data analysis.

**Supplemental Figure S2.Dose finding experiments of PBMCsec-pretreated basophils.** Basophils were pre-treated with two different doses of PBMCsec (8.33 U/mL and 12.5 U/mL) and the same volume of 0.9 % NaCl served as control. Basophils were treated with indicated stimuli and basophil activation was assessed by percentage of CD63-positive cells. Each symbol identifies one individual donor. Horizontal bars indicate arithmetic means and standard deviations.

**Supplemental Figure S3. Biological processes associated with genes upregulated by PBMCsec in skin mast cells.** Gene ontologies were calculated using 208 genes upregulated by PBMCsec compared to medium in skin mast cells. The GO term/pathway network connectivity Kappa score was set to 0.4. Each circle represents one GO term. Lines connect GO terms that share the same genes.

**Supplemental Figure S4. *In situ* mast cell degranulation in human skin.** Untreated (control)-, medium-, and PBMCsec-pretreated human skin biopsies were exposed to compound 48/80 and mast cells were stained by toluidine blue. Nuclei are stained blue, mast cells are violet. Closed arrow heads indicate non-degranulated mast cells, open arrow heads show degranulated mast cells. Scale bar 25 µm.

**Supplemental Figure S5**. **Viability of medium- and PBMCsec-treated basophils.** Basophil activation test was performed with medium- or 12.5 U/mL PBMCsec-pretreated basophils. Percentage of viable cells was determined by flow cytometry.

# **Supplemental Figure S6. Activation of PBMCsec- or medium-treated basophils from individuals allergic to birch pollens after Bet v 1 stimulation.** (a) Representative dot plots and histograms of control, medium-, and PBMCsec-treated basophils are shown. Lymphocytes and monocytes were gated in forward scatter (FSC) and side scatter (SSC) plots and basophils were identified based on CCR3 and CD123 expression. CD63 expression of CD123^+^, CCR3^+^ basophils was quantified. (b) Statistical analysis of CD63-expressing cells after stimulation with 10 ng/mL Bet v 1. Data of n=4 for medium and n=7 donors for control and PBMCsec are shown. Each symbol identifies an individual donor. Groups were compared by ordinary one-way ANOVA with Dunnett’s *post hoc* tests.

**Supplemental Figure S7. Activation of PBMCsec- or medium-treated basophils from individuals allergic to birch pollens after stimulation with 10 ng/mL Mal d 1**. (a) Representative dot plots and histograms of control, medium-, and PBMCsec-treated basophils are shown. Lymphocytes and monocytes were gated in forward scatter (FSC) and side scatter (SSC) plots and basophils were identified based on CCR3 and CD123 expression. CD63 expression of CD123^+^, CCR3^+^ basophils was quantified. (b) Statistical analysis of CD63-expressing cells after stimulation with 10 ng/mL Mal d 1. Data of n=4 donors per treatment condition are shown. Each symbol identifies an individual donor. Groups were compared by ordinary one-way ANOVA with Dunnett’s *post hoc* tests.

**Supplemental Figure S8. Activation of PBMCsec- or medium-treated basophils from individuals allergic to birch pollens after stimulation with 100 ng/mL Mal d 1.** (a) Representative dot plots and histograms of control, medium-, and PBMCsec-treated basophils are shown. Lymphocytes and monocytes were gated in forward scatter (FSC) and side scatter (SSC) plots and basophils were identified based on CCR3 and CD123 expression. CD63 expression of CD123^+^, CCR3^+^ basophils was quantified. (b) Statistical analysis of CD63-expressing cells after stimulation with 100 ng/mL Mal d 1. Data of n=4 donors per treatment condition are shown. Each symbol identifies an individual donor. Groups were compared by ordinary one-way ANOVA with Dunnett’s *post hoc* tests.

# **Supplemental Figure S9. Activation of PBMCsec- or medium-treated basophils from individuals allergic to birch pollens after stimulation with fMLP.** (a) Representative dot plots and histograms of control, medium-, and PBMCsec-treated basophils are shown. Lymphocytes and monocytes were gated in forward scatter (FSC) and side scatter (SSC) plots and basophils were identified based on CCR3 and CD123 expression. CD63 expression of CD123^+^, CCR3^+^ basophils was quantified. (b) Statistical analysis of CD63-expressing cells after stimulation with fMLP. Data of n=11 donors for controls, n=8 for medium, and n=15 for PBMCsec are shown. Each symbol identifies an individual donor. Groups were compared by ordinary one-way ANOVA with Dunnett’s *post hoc* tests.

# **Supplemental Figure S10. Effect of PBMCsec on basophil gene expression signature.** Genes involved in neutrophil degranulation, Fc receptor and IL-1 signaling, and antigen presentation are shown. Bars indicate arithmetric means of log_2_-transformed expression values ± s.e.m. Data of n=3 donors are shown.

# **Supplemental Figure S11. Effect of PBMCsec on Fc-RI protein levels.** Fc-RI surface levels of human basophils was assessed after 1 hour pre-incubation with PBMCsec or medium by flow cytometry. Following pre-incubation, cells were either left untreated (control) or stimulated as indicated. Data of one representative donor of four are shown.

# **Supplemental Figure S12. Fc-RI expression levels after long-time exposure to PBMCsec.** Fc-RI expression of human basophils was assessed after 24 hours incubation with PBMCsec or medium by flow cytometry. Data of five individual donors are shown.

# **Supplemental Figure S13. LYN phosphorylation status of PBMCsec-treated basophils after Bet v 1 stimulation.** Representative histograms of phosphorylated LYN of PBMCsec- or medium-pre-treated human basophils after Bet v 1 stimulation. Phosphorylation levels were assessed at indicated time points after stimulation.

**Supplemental Figure S14.** **CD300A surface expression of human basophils.** CD300A surface expression was assessed by flow cytometry on medium-, PBMCsec-, and lipid-treated human basophils. Representative data of n=3 donors are shown.

# **Supplemental Figure S15. Effect of PBMCsec on gene expression of inhibitory surface molecules.** Bars indicate arithmetric means of log_2_-transformed expression values ± s.e.m. Data of n=3 donors are shown.

# **Supplemental Figure S16. Lipids present in PBMCsec abrogate Bet v 1-driven basophil activation.** Basophils were pre-treated with medium, PBMCsec, or lipids and basophil activation was induced by 10 ng/mL Bet v 1. Data of n=4 donors were compared by one-way ANOVA with Dunnett’s *post hoc* tests. Each symbol identifies an individual donor.

# **Supplemental Figure S17. Annexin V-mediated blockade of PS in PBMCsec.** PBMCsec and medium were pre-incubated with annexin V and CaCl_2_ before addition to basophils. Then, BATs were performed after stimulation with anti-IgE or Bet v 1 (1 ng/mL or 10 ng/mL).

# **Supplemental Figure S18. Neutralizing antibody-mediated blockade of PS in PBMCsec-derived lipids.** Lipids and medium were pre-incubated with two different concentrations of neutralizing anti-PS antibody (50 µg/mL and 100 µg/mL). Basophils were then pre-treated with medium or lipids and BATs were performed with 1 ng/mL Bet v 1.

**Supplemental Figure S19. Blockade of CD300A by neutralizing antibodies.** Medium or lipids were pre-incubated with anti-CD300A neutralizing antibodies. Basophils were then pre-treated with medium or lipids and BATs were performed with 0.1 ng/mL Bet v 1. Each symbol identifies one individual donor.

# **Supplemental Figure S20. Mass spectrometric quantification of lipid species in medium and PBMCsec.** LPE lysophosphatidylethanolamine, PC phosphatidylcholine, PE phosphatidylethanolamine.
