# Supplementary figures and images for: The secretome of irradiated peripheral blood mononuclear cells attenuates activation of mast cells and basophils
Source: eBioMedicine. 2022 Jun 4;81:104093. doi: 10.1016/j.ebiom.2022.104093 (PMC9168057; doi:10.1016/j.ebiom.2022.104093)

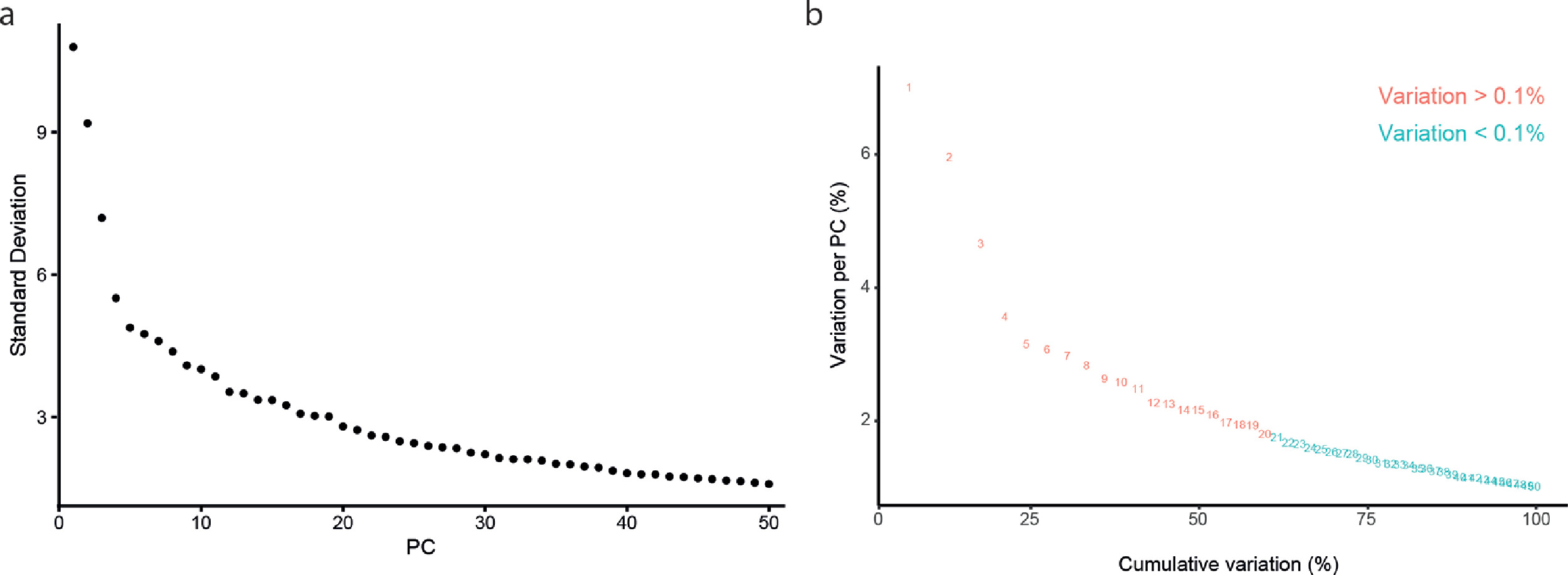

Supplement: Supplementary file 2 [file mmc2.jpg]

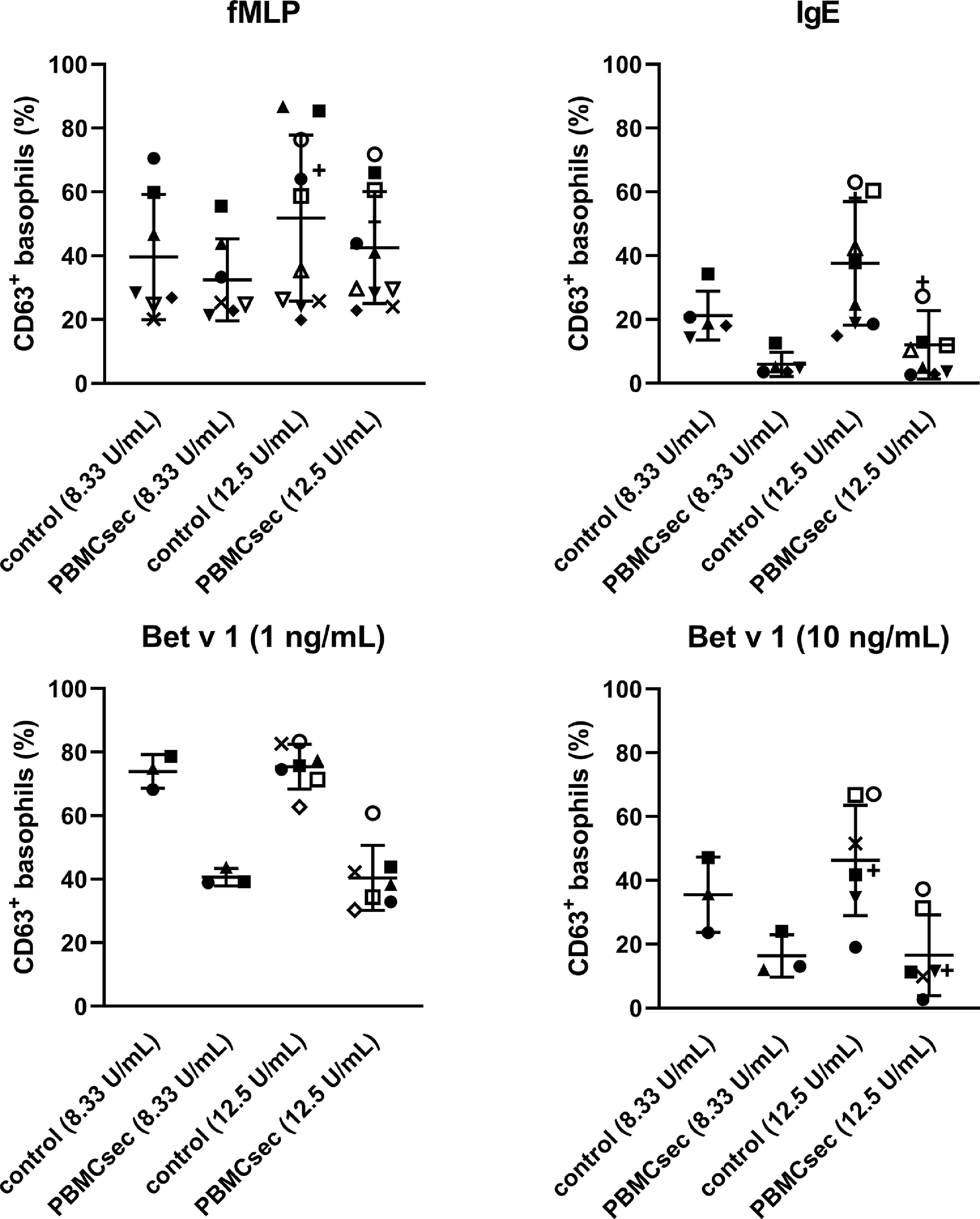

Supplement: Supplementary file 3 [file mmc3.jpg]

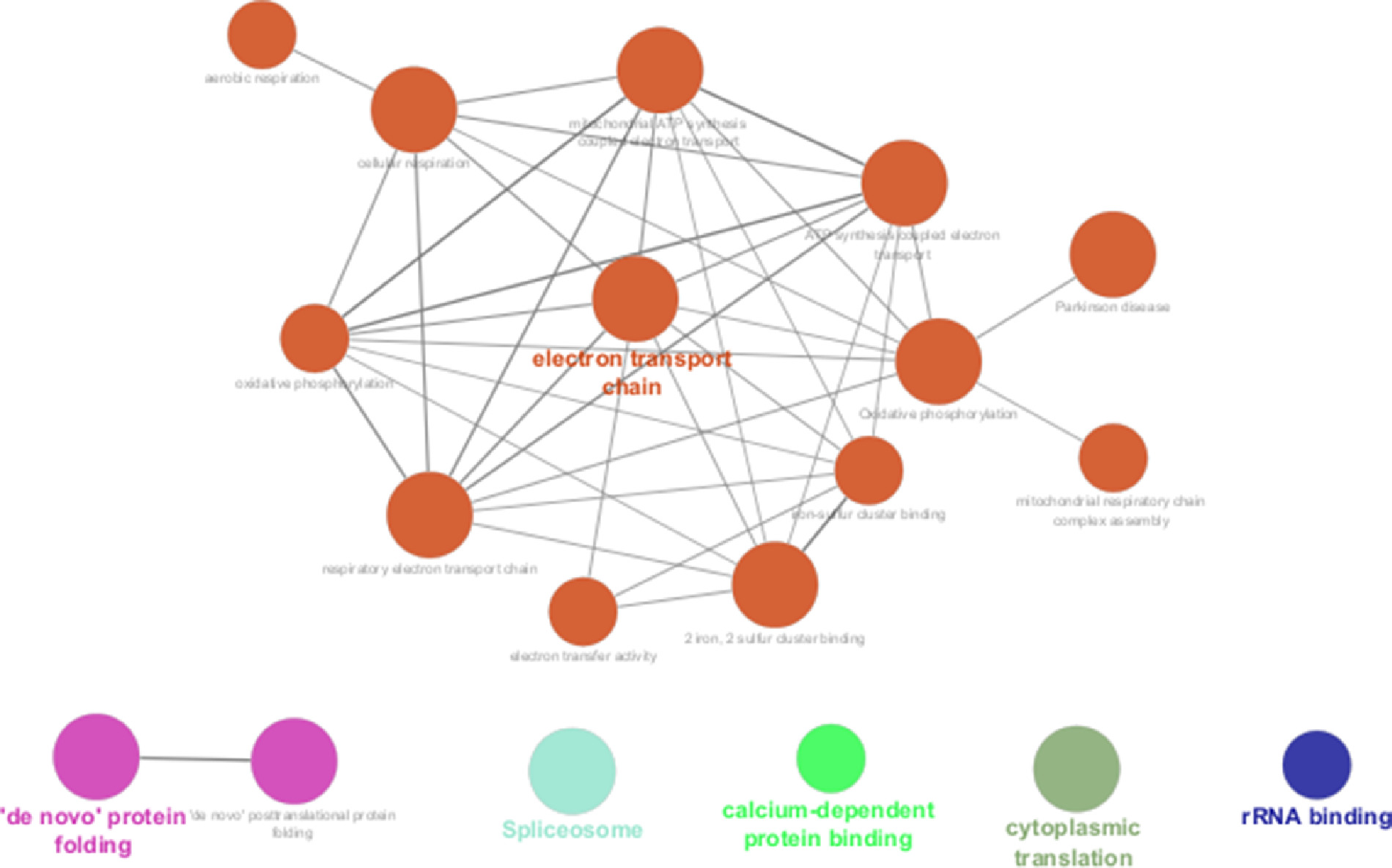

Supplement: Supplementary file 4 [file mmc4.jpg]

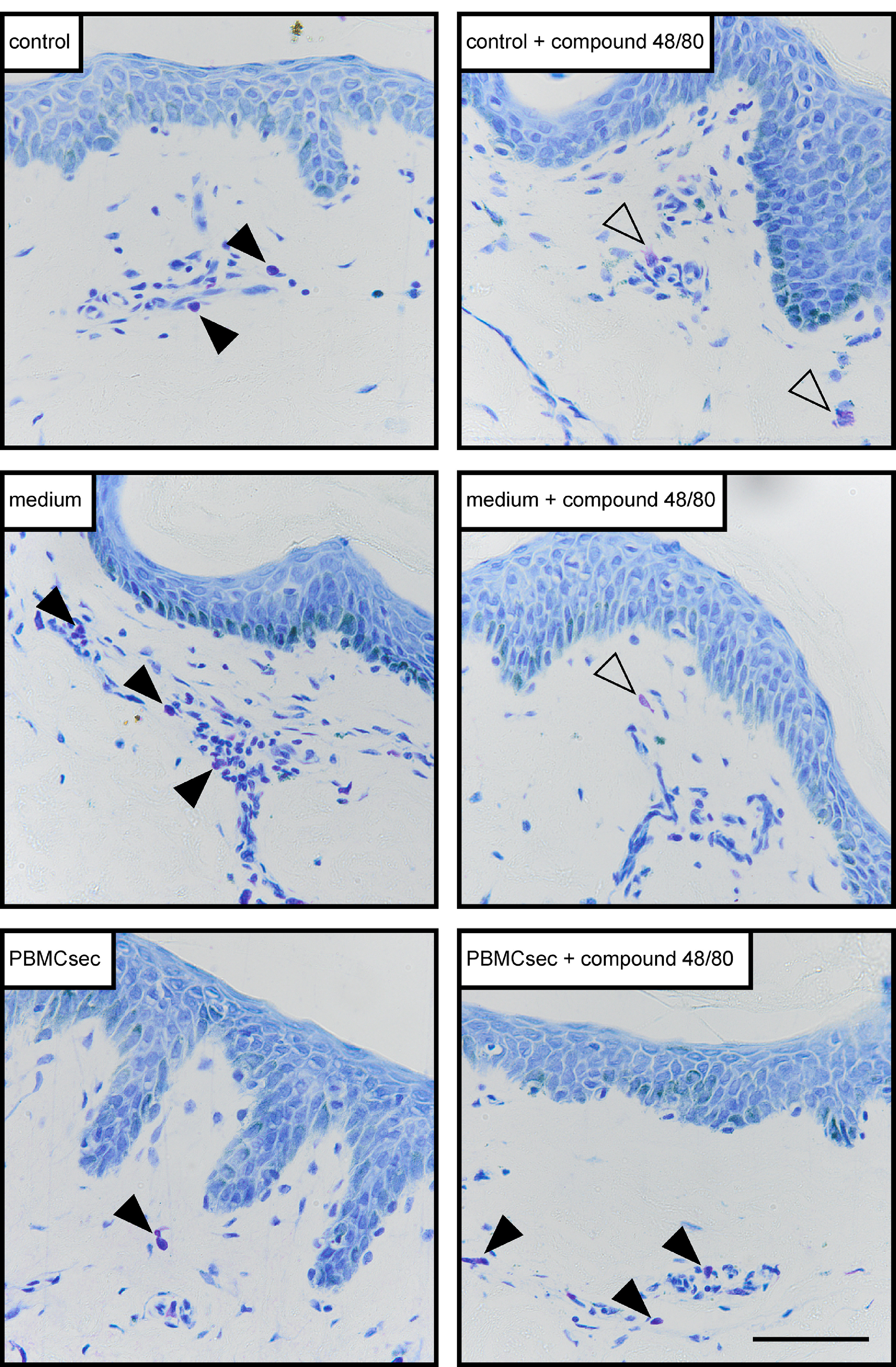

Supplement: Supplementary file 5 [file mmc5.jpg]

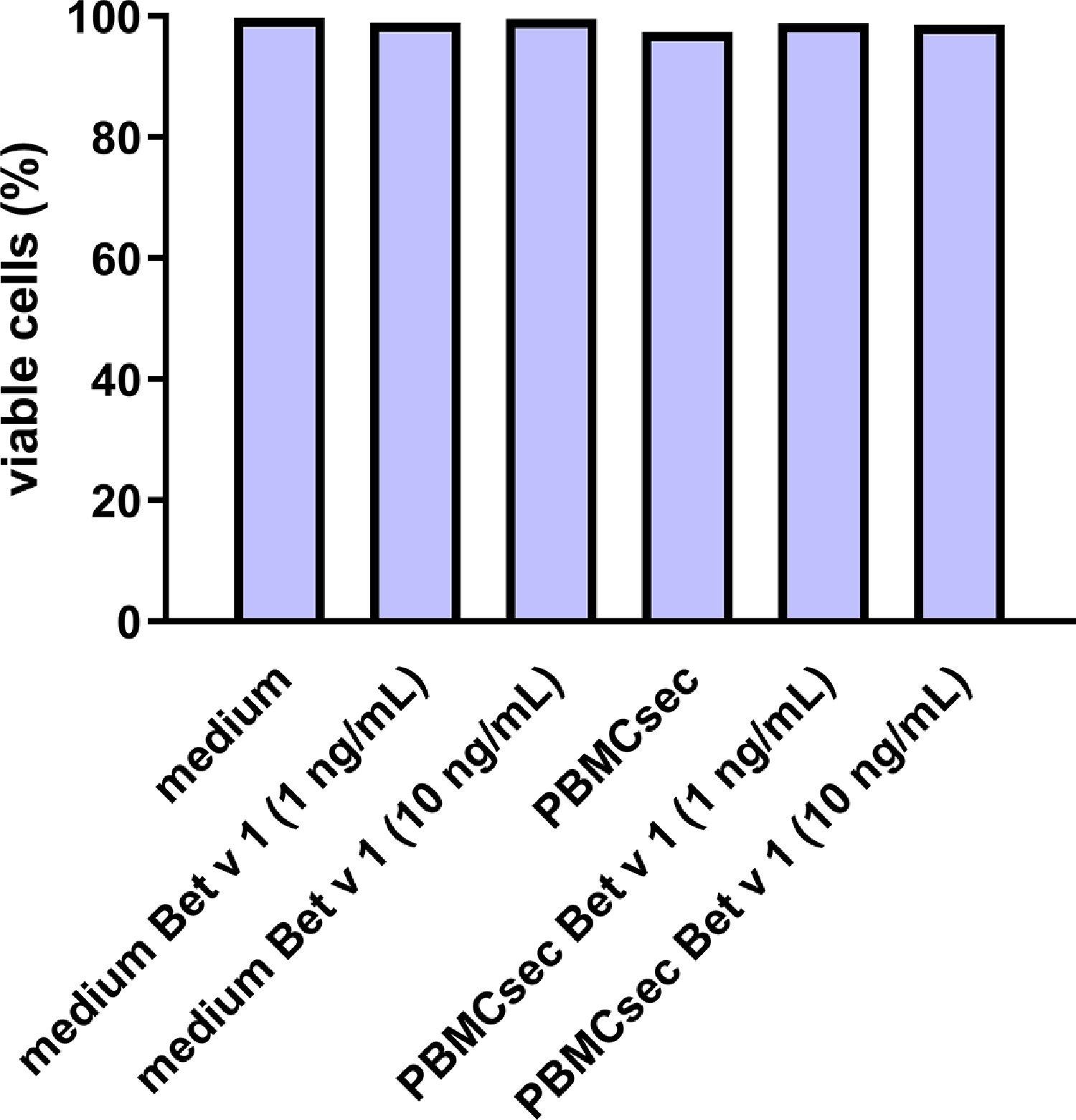

Supplement: Supplementary file 6 [file mmc6.jpg]

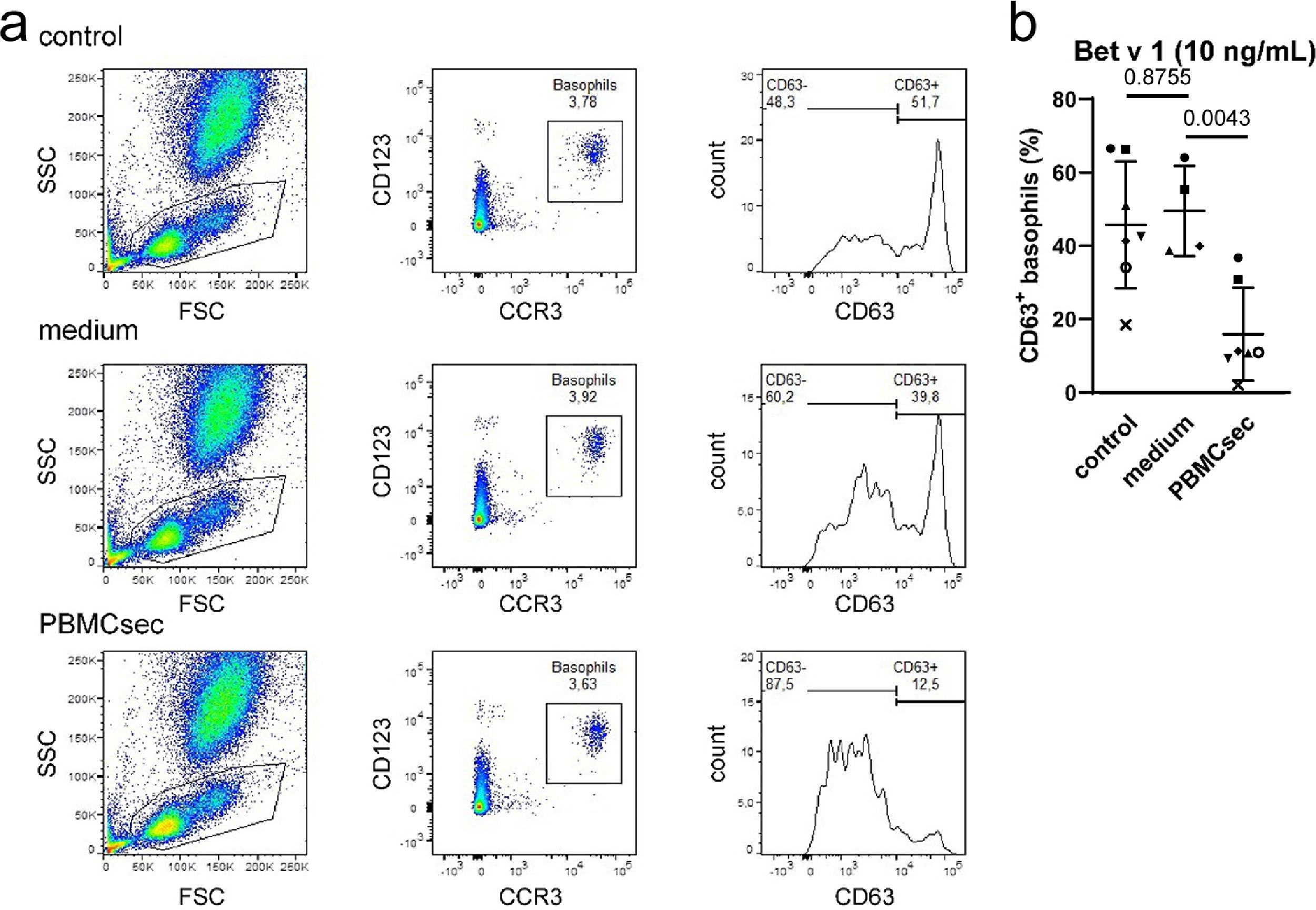

Supplement: Supplementary file 7 [file mmc7.jpg]

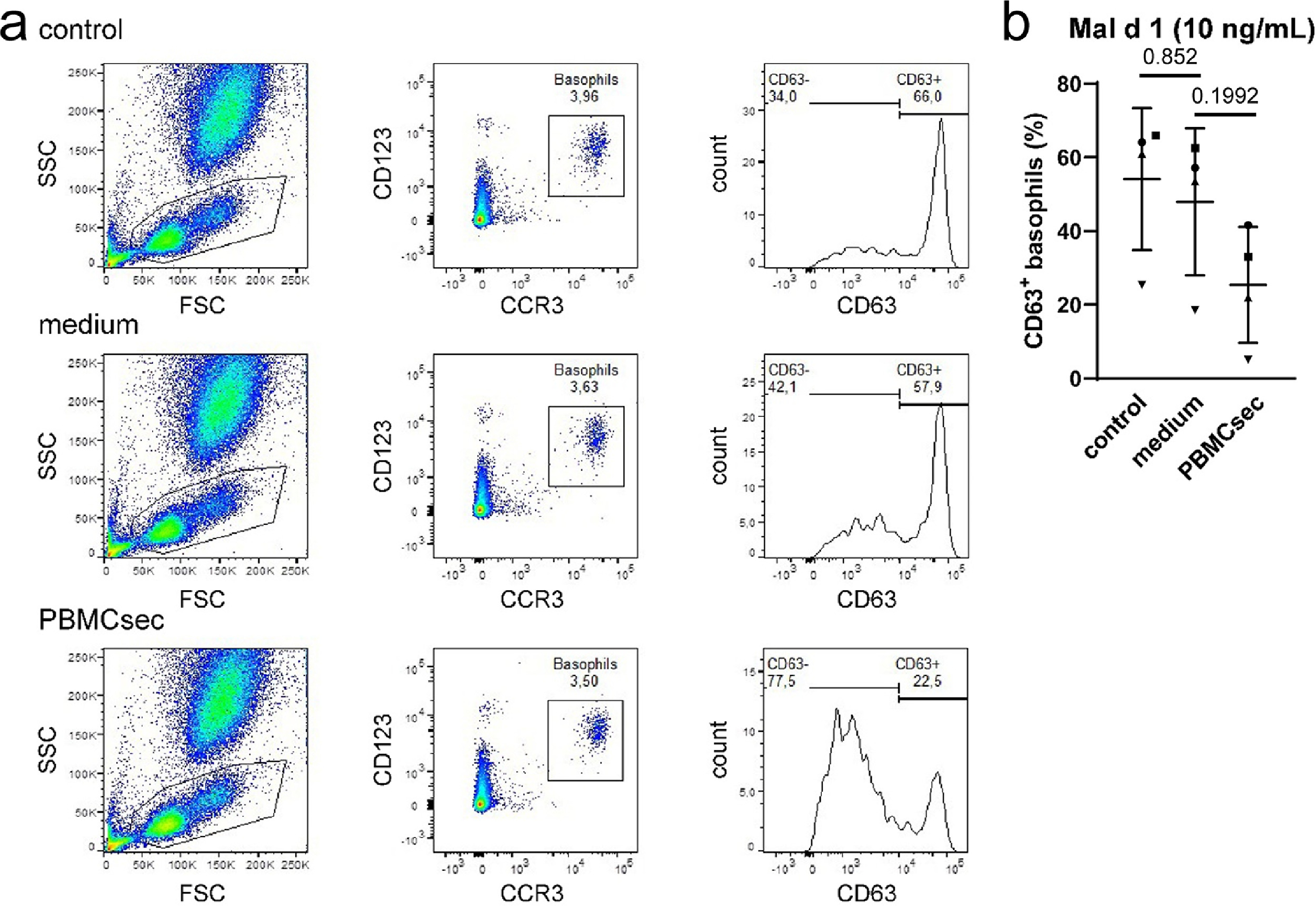

Supplement: Supplementary file 8 [file mmc8.jpg]

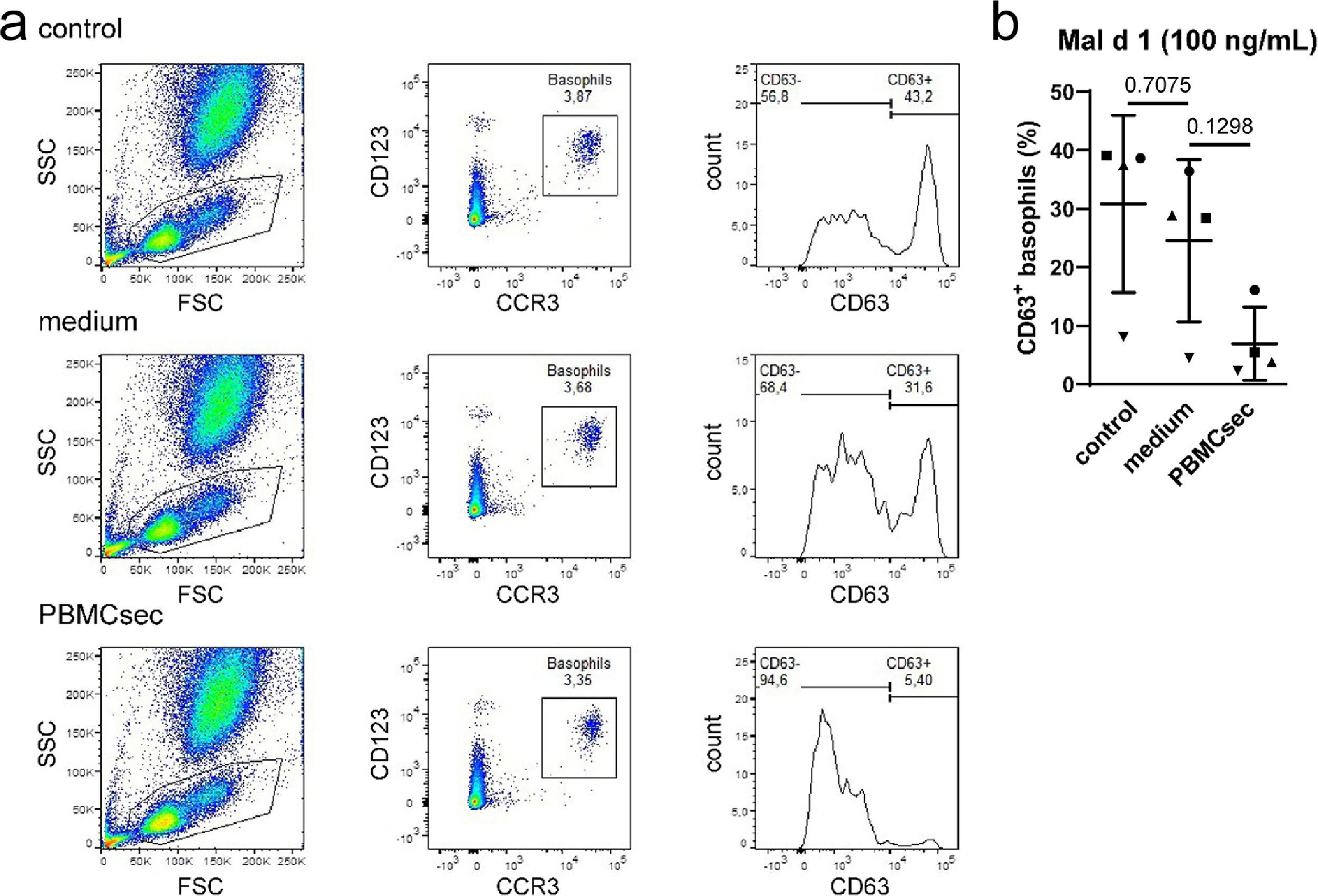

Supplement: Supplementary file 9 [file mmc9.jpg]

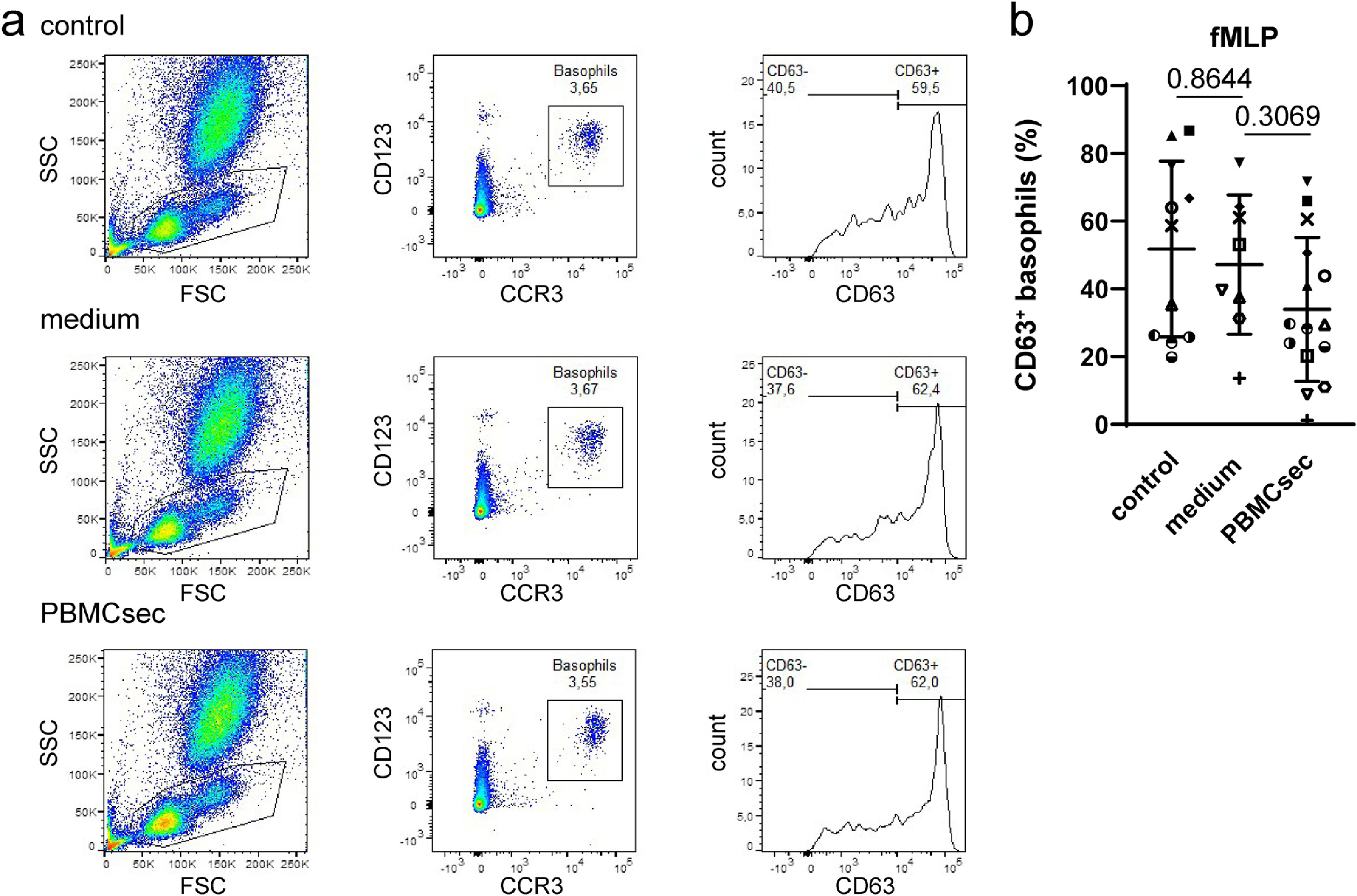

Supplement: Supplementary file 10 [file mmc10.jpg]

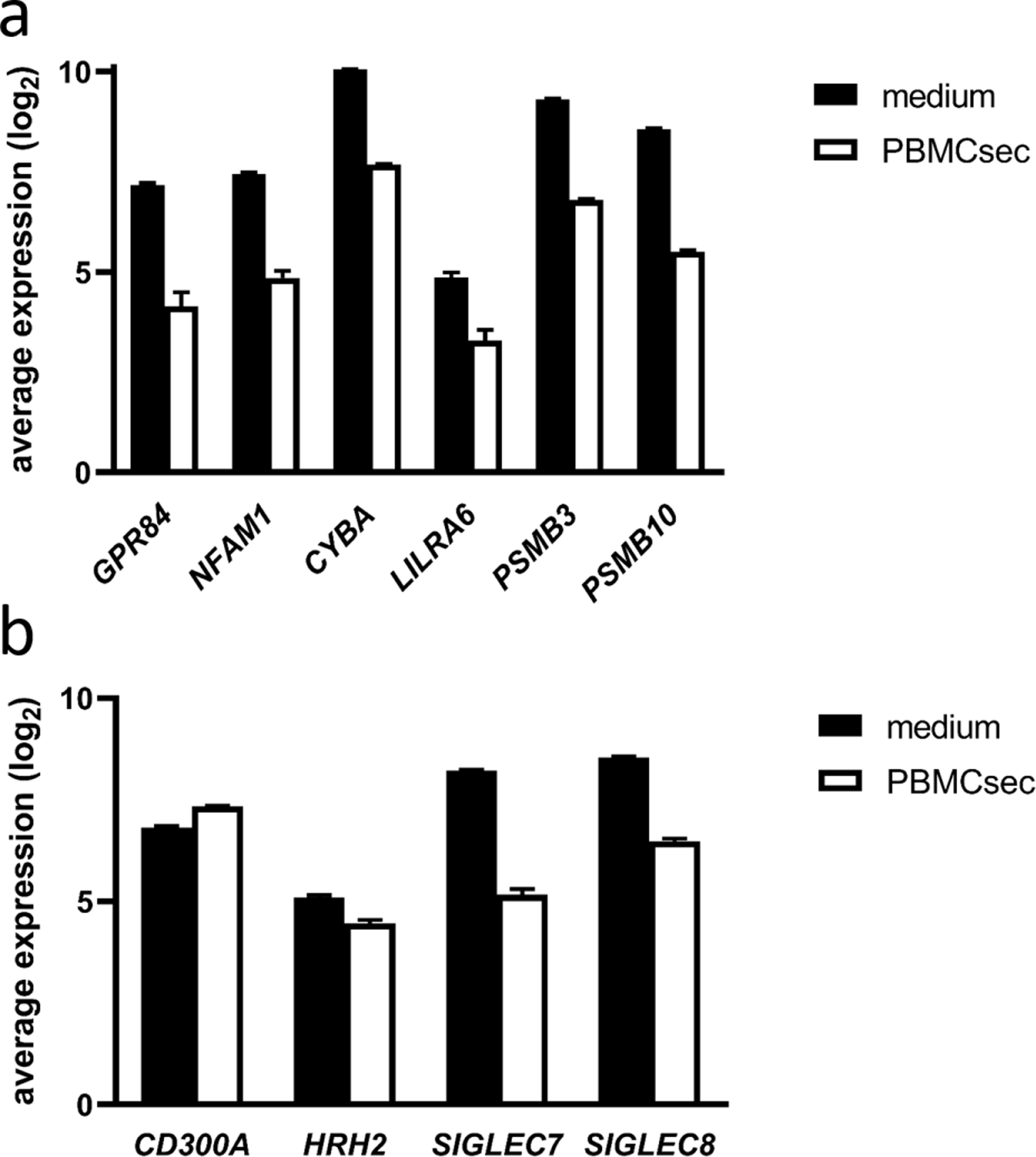

Supplement: Supplementary file 11 [file mmc11.jpg]

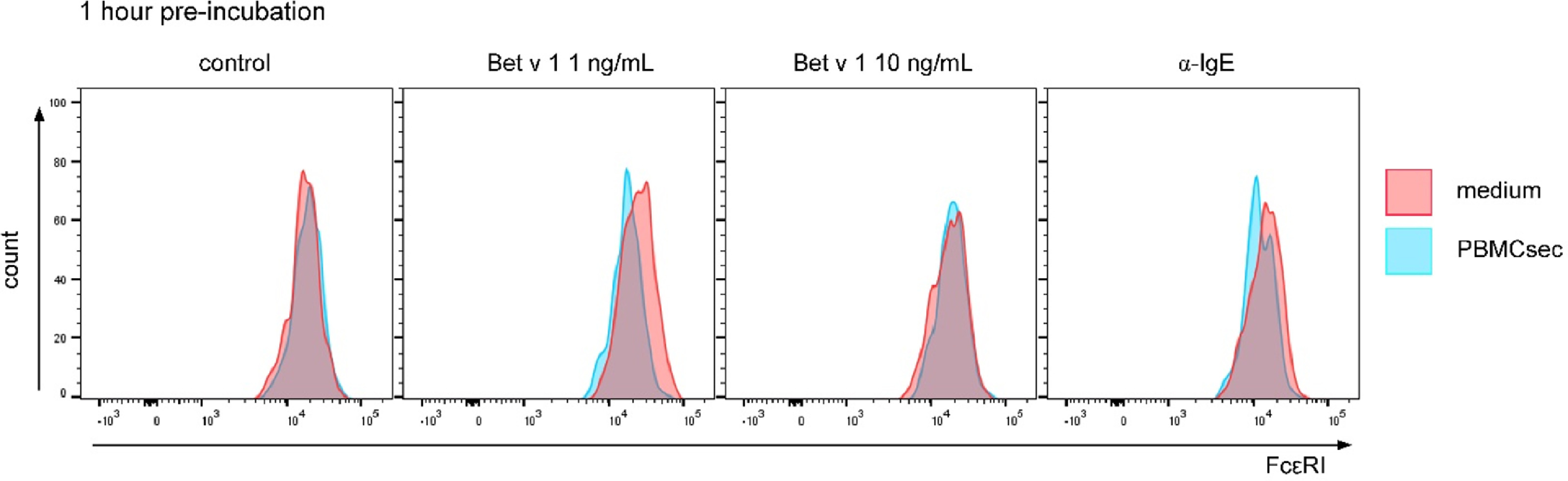

Supplement: Supplementary file 12 [file mmc12.jpg]

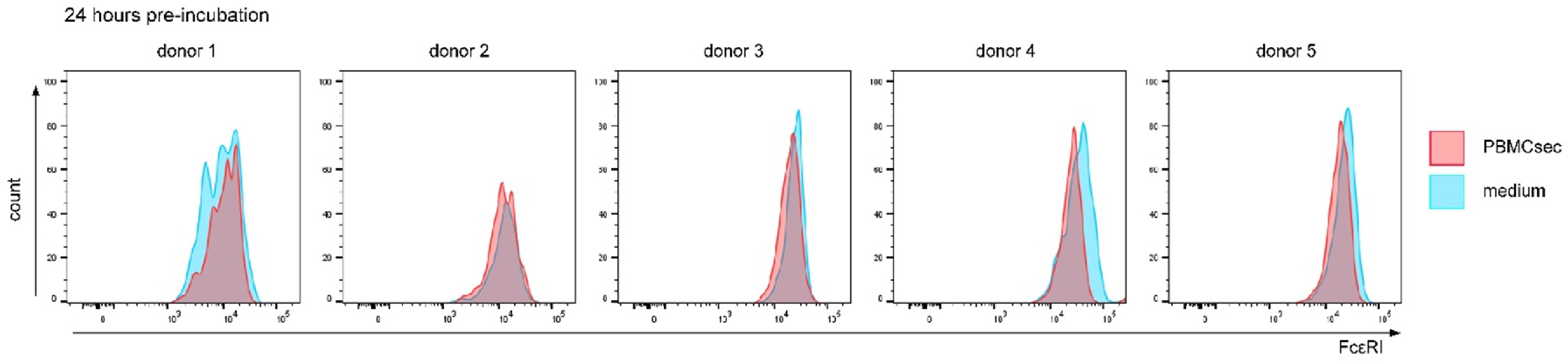

Supplement: Supplementary file 13 [file mmc13.jpg]

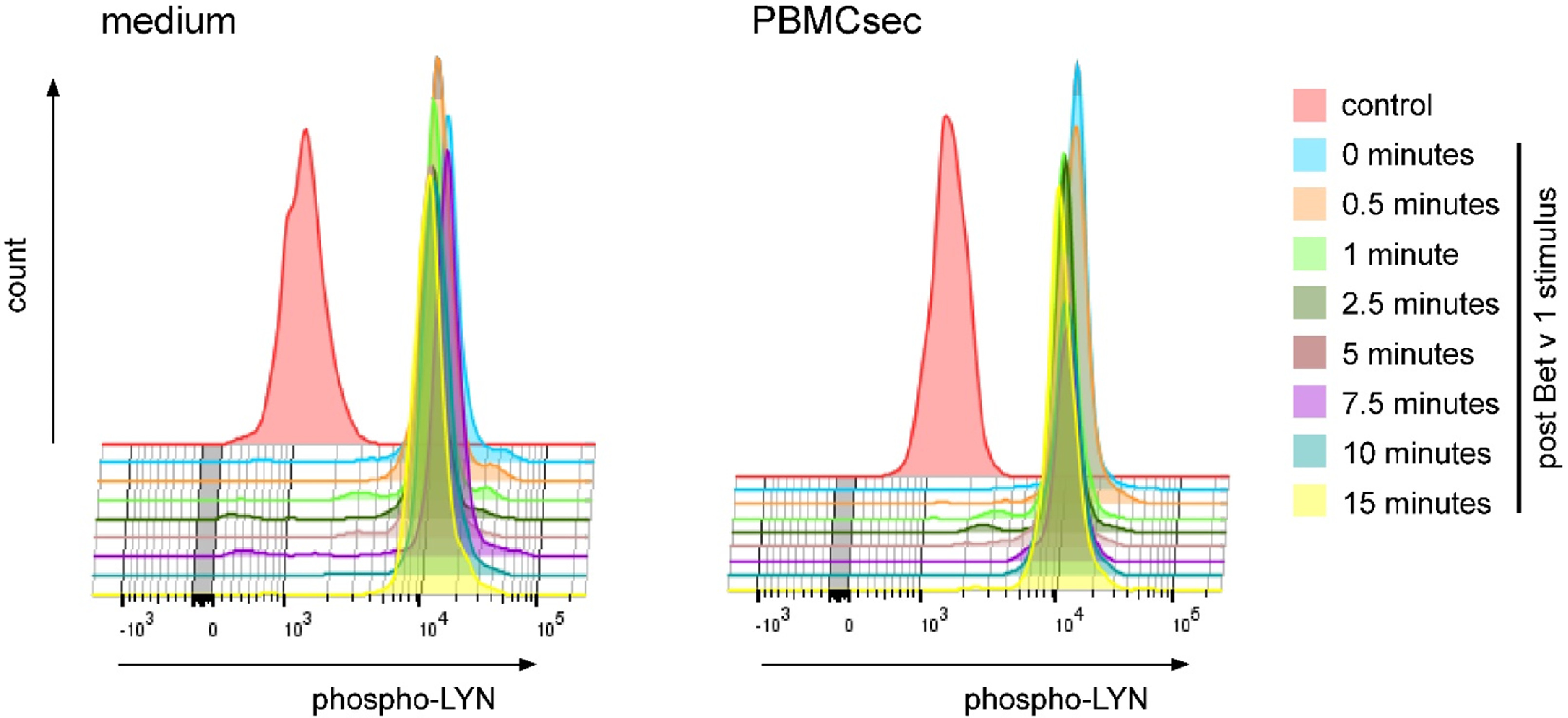

Supplement: Supplementary file 14 [file mmc14.jpg]

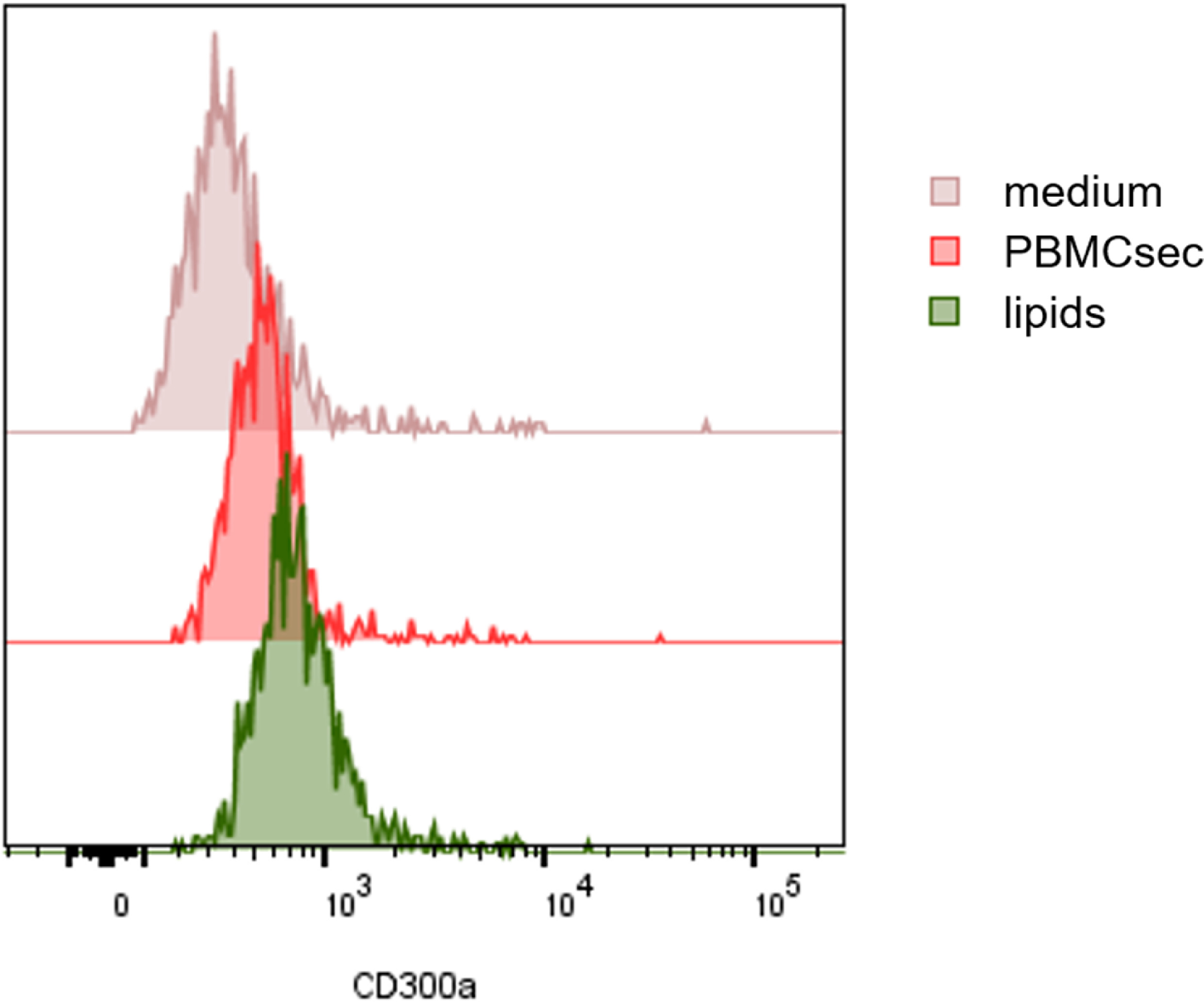

Supplement: Supplementary file 15 [file mmc15.jpg]

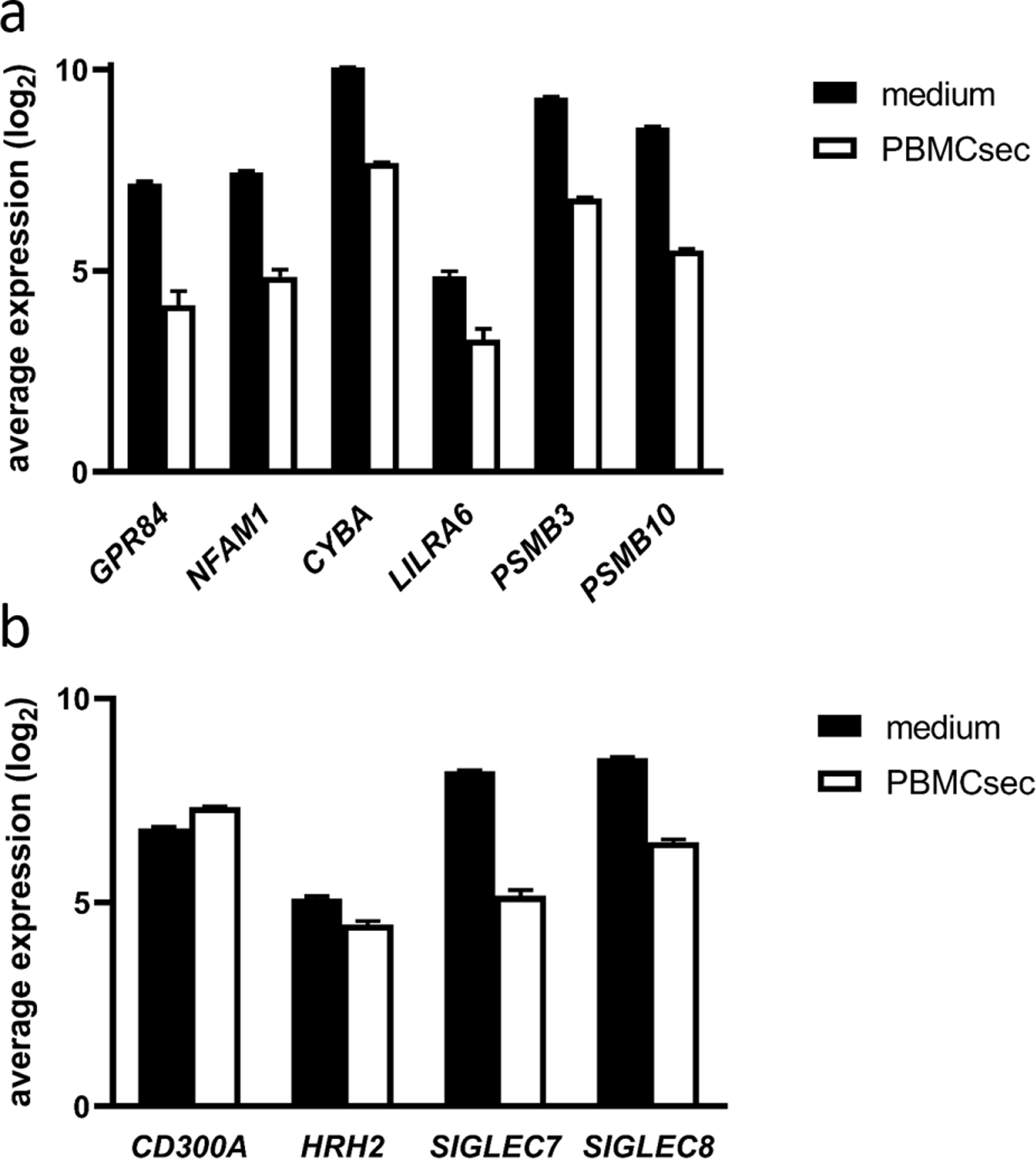

Supplement: Supplementary file 16 [file mmc16.jpg]

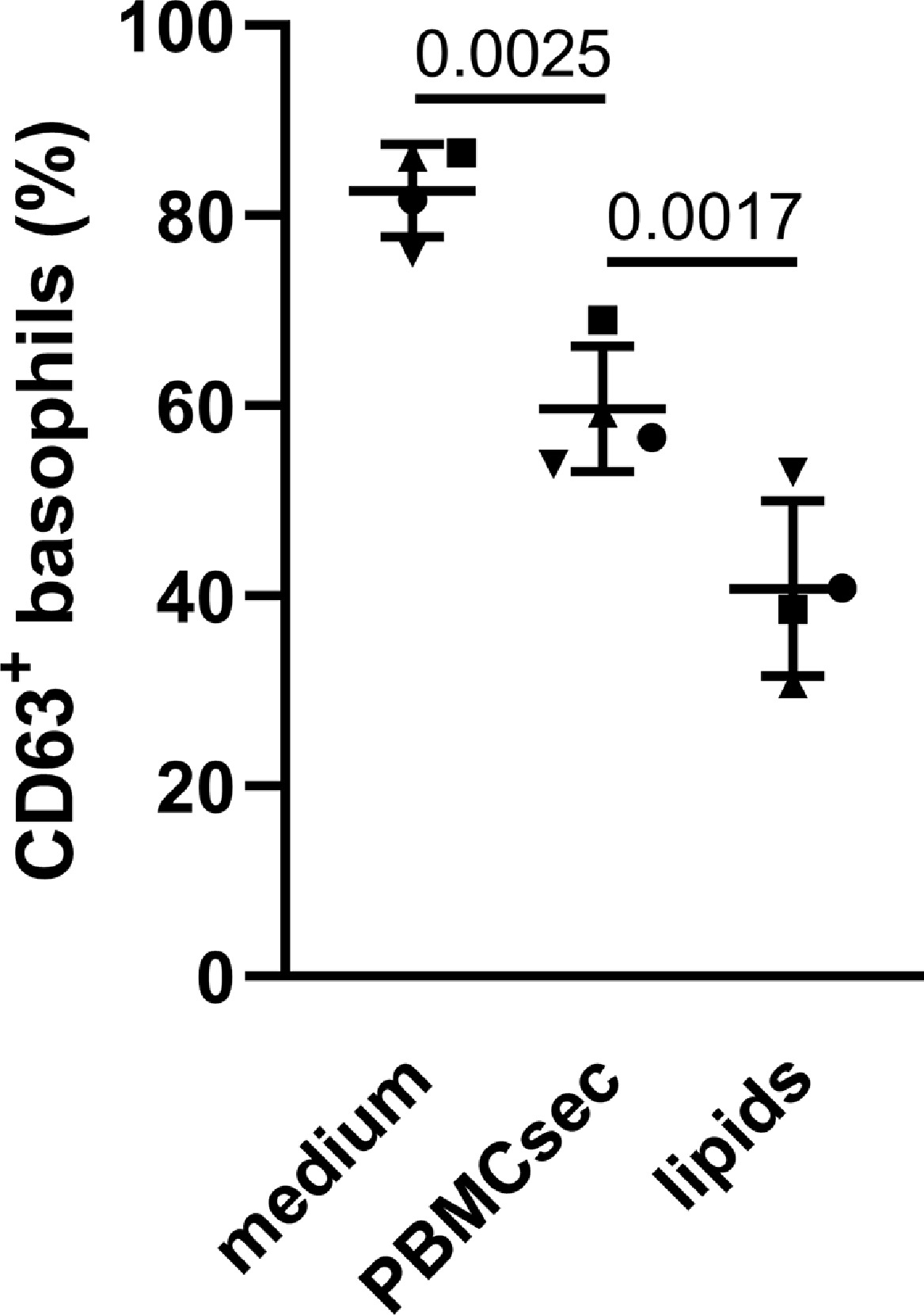

Supplement: Supplementary file 17 [file mmc17.jpg]

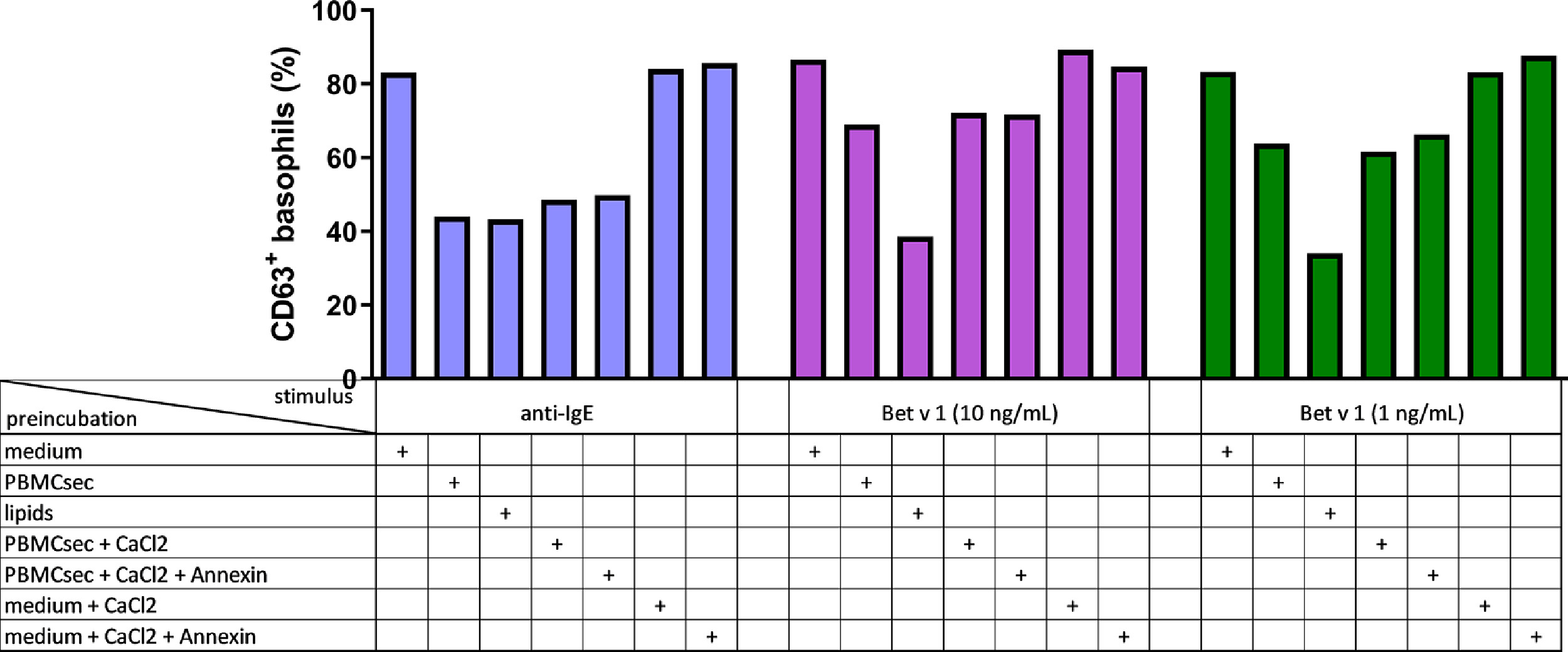

Supplement: Supplementary file 18 [file mmc18.jpg]

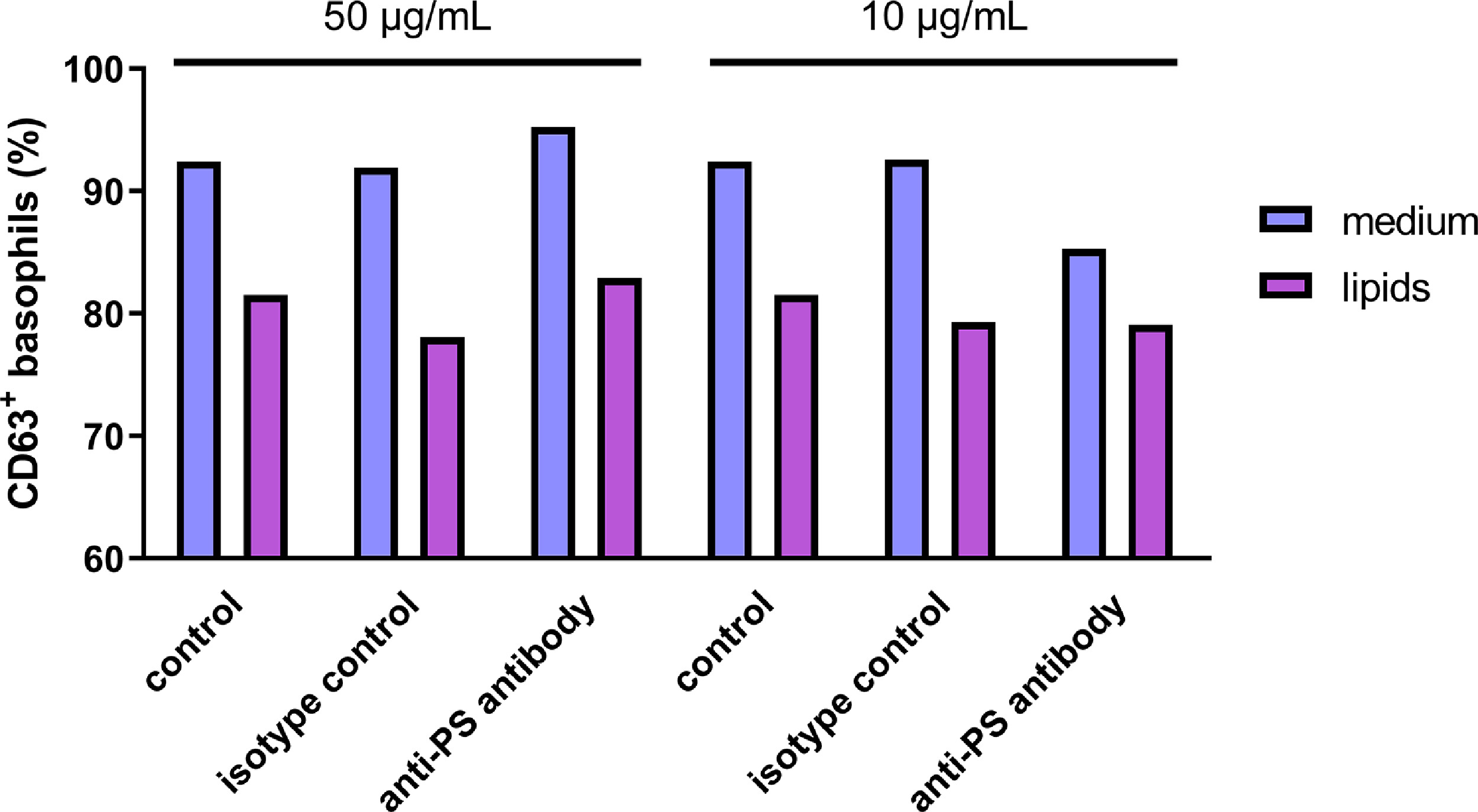

Supplement: Supplementary file 19 [file mmc19.jpg]

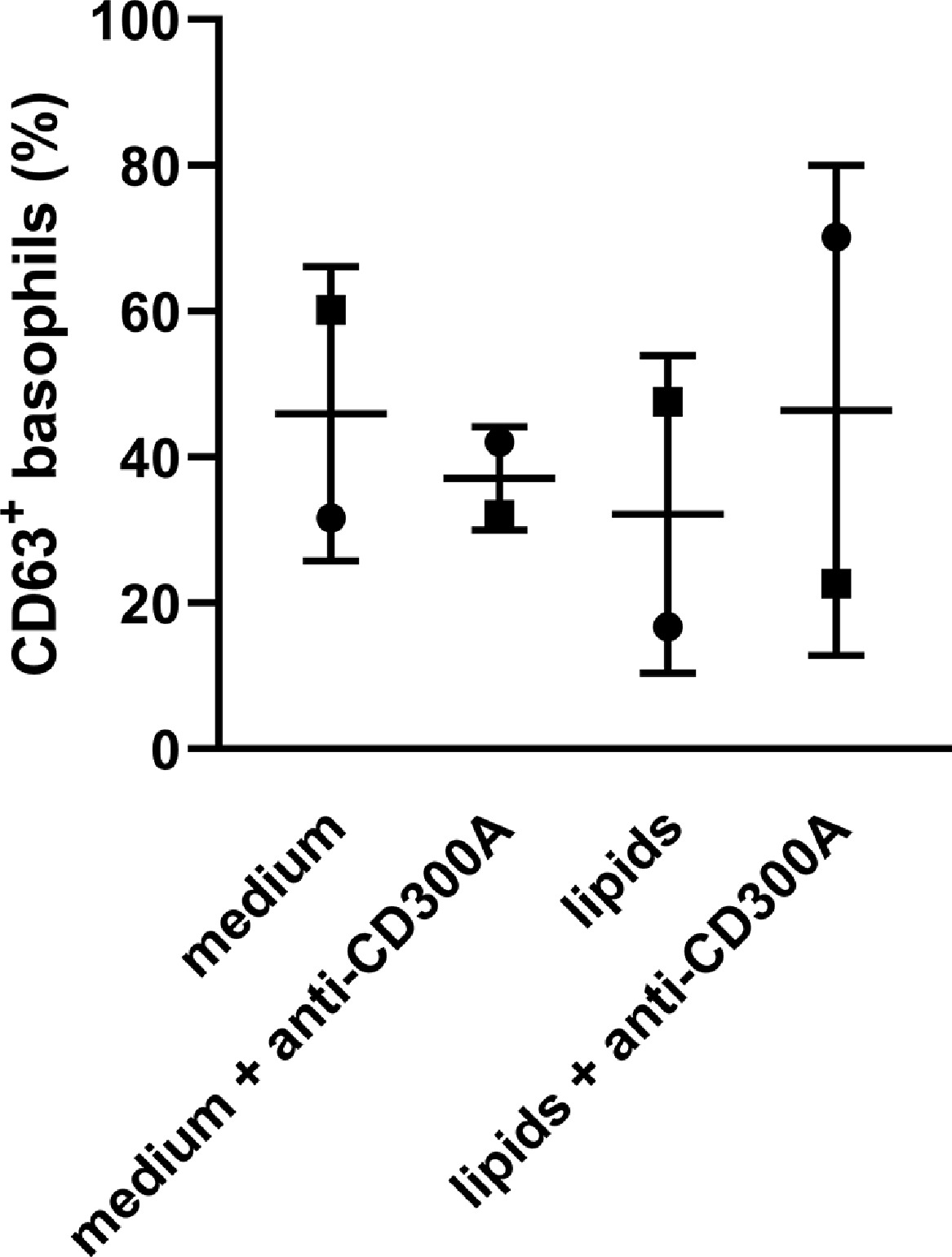

Supplement: Supplementary file 20 [file mmc20.jpg]

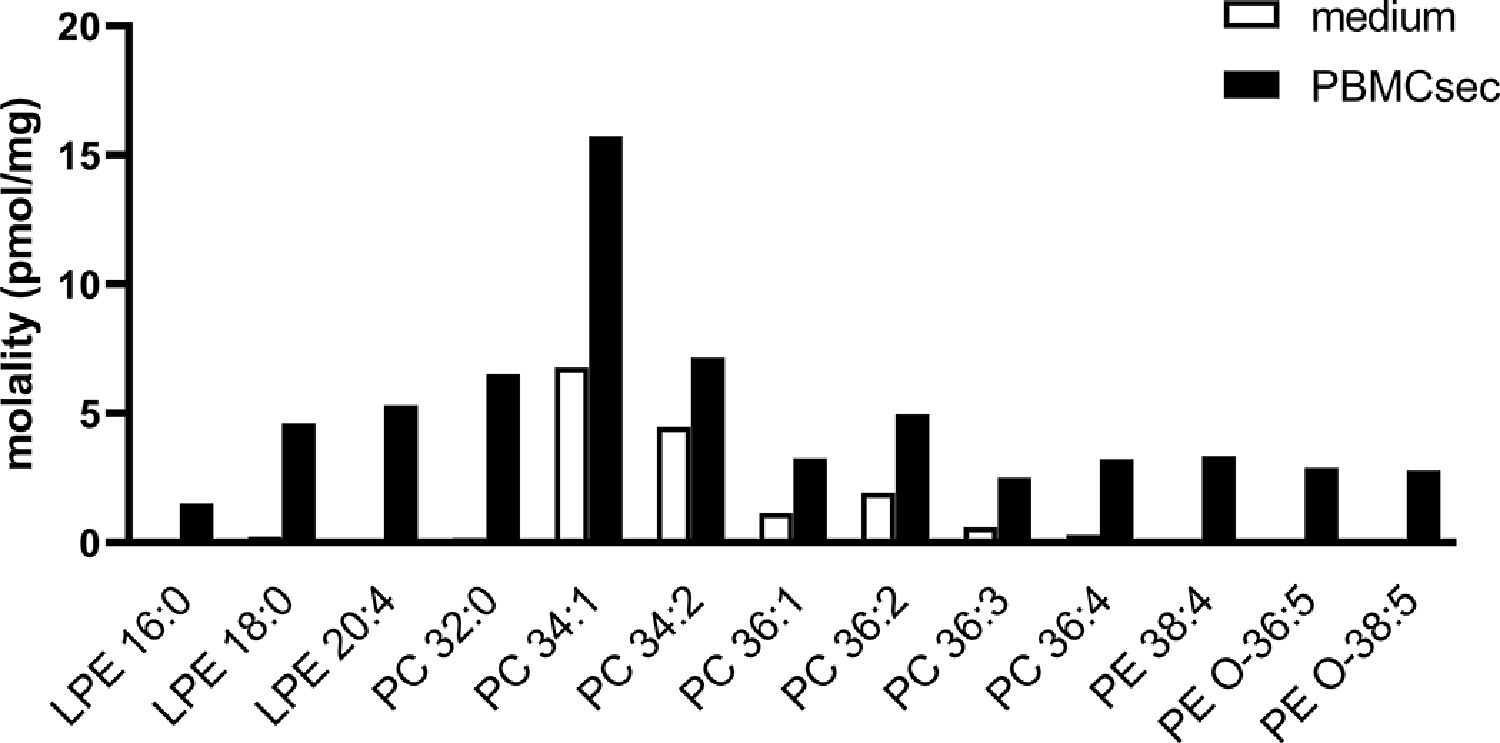

Supplement: Supplementary file 21 [file mmc21.jpg]
