# Supplementary material for: The secretome of irradiated peripheral blood mononuclear cells attenuates activation of mast cells and basophils
Source: eBioMedicine. 2022 Jun 4;81:104093. doi: 10.1016/j.ebiom.2022.104093 (PMC9168057; doi:10.1016/j.ebiom.2022.104093)
Supplement: Supplementary file 22 [file mmc22.docx]

**Supplemental Table S1.** Averaged, log_2_-transformed expressions of arachidonate lipoxygenases, cytochrome c oxydases, or leukotriene- and prostaglandin-associated genes. Data were statistically analyzed by empirical Bayes method and p values < 0.05 were highlighted in bold.

|  | gene symbol | PBMCsec (avg log_2_) | medium (avg log_2_) | fold change | p-val | description |
| --- | --- | --- | --- | --- | --- | --- |
| alox gene family | *ALOX5* | 8.61 | 8.97 | -1.28 | 0.5772 | arachidonate 5-lipoxygenase |
|  | *ALOX5AP* | 11.43 | 11.59 | -1.12 | 0.4712 | arachidonate 5-lipoxygenase-activating protein |
|  | *ALOX12* | 2.37 | 2.75 | -1.3 | 0.5595 | arachidonate 12-lipoxygenase |
|  | *ALOX12B* | 2.06 | 2.98 | -1.9 | 0.066 | arachidonate 12-lipoxygenase, 12R type |
|  | *ALOX15* | 5.21 | 5.66 | -1.36 | 0.6812 | arachidonate 15-lipoxygenase |
|  | *ALOX15B* | 2.67 | 2.76 | -1.07 | 0.9015 | arachidonate 15-lipoxygenase, type B |
|  | *ALOXE3* | 2.4 | 2.65 | -1.19 | 0.7662 | arachidonate lipoxygenase 3 |
| cytochrome c oxidase gene family | *COX1* | 10.72 | 10.4 | 1.24 | 0.0849 | cytochrome c oxidase subunit I |
|  | *COX2* | 8.95 | 8.74 | 1.16 | 0.2113 | cytochrome c oxidase subunit II |
|  | *COX4I1* | 9.2 | 10.46 | -2.4 | 0.7262 | cytochrome c oxidase subunit IV isoform 1 |
|  | *COX4I2* | 2.94 | 3.27 | -1.26 | 0.9458 | cytochrome c oxidase subunit IV isoform 2 (lung) |
|  | *COX5A* | 6.64 | 4.21 | 5.39 | 0.0757 | cytochrome c oxidase subunit Va |
|  | *COX5B* | 3 | 5.08 | -4.22 | 0.091 | cytochrome c oxidase subunit Vb |
|  | *COX6A1* | 2.25 | 1.73 | 1.43 | 0.9307 | cytochrome c oxidase subunit VIa polypeptide 1 |
|  | *COX6A2* | 3.68 | 4.91 | -2.35 | 0.0769 | cytochrome c oxidase subunit VIa polypeptide 2 |
|  | *COX6B1* | 7.37 | 7.71 | -1.26 | 0.7573 | cytochrome c oxidase subunit VIb polypeptide 1 (ubiquitous) |
|  | *COX6B2* | 2.38 | 3.16 | -1.71 | 0.0999 | cytochrome c oxidase subunit VIb polypeptide 2 (testis) |
|  | *COX6C* | 3.53 | 3.84 | -1.24 | 0.8956 | cytochrome c oxidase subunit VIc |
|  | *COX7A1* | 2.59 | 2.74 | -1.11 | 0.6646 | cytochrome c oxidase subunit VIIa polypeptide 1 (muscle) |
|  | *COX7A2* | 6.12 | 7.41 | -2.44 | 0.7214 | cytochrome c oxidase subunit VIIa polypeptide 2 (liver) |
|  | *COX7B* | 4.72 | 5.19 | -1.39 | 0.7003 | cytochrome c oxidase subunit VIIb |
|  | *COX7B2* | 2.52 | 2.52 | 1 | 0.9925 | cytochrome c oxidase subunit VIIb2 |
|  | *COX7C* | 6.22 | 6.23 | -1.01 | 0.9678 | cytochrome c oxidase subunit VIIc |
|  | *COX8A* | 8.83 | 10.14 | -2.48 | 0.631 | cytochrome c oxidase subunit VIIIA (ubiquitous) |
|  | *COX8C* | 1.99 | 1.96 | 1.02 | 0.9013 | cytochrome c oxidase subunit VIIIC |
|  | *COX10* | 3.46 | 3.68 | -1.17 | 0.8204 | COX10 heme A:farnesyltransferase cytochrome c oxidase assembly factor |
|  | *COX11* | 3.43 | 3.61 | -1.13 | 0.3934 | COX11 cytochrome c oxidase copper chaperone |
|  | *COX14* | 4.01 | 4.84 | -1.77 | 0.2586 | COX14 cytochrome c oxidase assembly factor |
|  | *COX15* | 4.1 | 5.86 | -3.38 | 0.1402 | cytochrome c oxidase assembly homolog 15 (yeast) |
|  | *COX17* | 4.83 | 4.66 | 1.12 | 0.5892 | COX17 cytochrome c oxidase copper chaperone |
|  | *COX18* | 3.96 | 4.02 | -1.04 | 0.9176 | COX18 cytochrome c oxidase assembly factor |
|  | *COX19* | 5.01 | 3.53 | 2.78 | 0.099 | COX19 cytochrome c oxidase assembly factor |
|  | *COX20* | 5.39 | 4.11 | 2.43 | 0.1687 | COX20 cytochrome c oxidase assembly factor |

| Supplemental Table S1, continued. | | | | | | |
| --- | --- | --- | --- | --- | --- | --- |
|  | gene symbol | PBMCsec (avg log_2_) | medium (avg log_2_) | fold change | p-val | description |
| leukotriene-associated  genes | *LTB* | 2.72 | 3.36 | -1.55 | 0,1534 | lymphotoxin beta (TNF superfamily, member 3) |
|  | *LTB4R* | 4.28 | 5.6 | -2.49 | 0,9205 | leukotriene B4 receptor |
|  | *LTB4R2* | 3.92 | 3.91 | 1.01 | 0,3112 | leukotriene B4 receptor 2 |
|  | *LTB4R; LTB4R2* | 4.1 | 4.11 | -1.01 | 0,4981 | leukotriene B4 receptor; leukotriene B4 receptor 2 |
|  | *LTBP1* | 3.93 | 3.48 | 1.37 | 0,372 | latent transforming growth factor beta binding protein 1 |
|  | *LTBP2* | 4.29 | 4.29 | 1 | 0,3146 | latent transforming growth factor beta binding protein 2 |
|  | *LTBP3* | 2.64 | 3.34 | -1.63 | 0,3564 | latent transforming growth factor beta binding protein 3 |
|  | *LTBP4* | 3.05 | 3.43 | -1.31 | 0,8118 | latent transforming growth factor beta binding protein 4 |
|  | *LTBR* | 3.95 | 5.04 | -2.12 | 0,645 | lymphotoxin beta receptor (TNFR superfamily, member 3) |
| prostaglandin-associated genes | *PTGDR* | 2.47 | 2.5 | -1.02 | 0,9758 | prostaglandin D2 receptor (DP) |
|  | *PTGDR2* | 5.67 | 5.07 | 1.51 | 0,4992 | prostaglandin D2 receptor 2 |
|  | *PTGDS* | 3.54 | 4.1 | -1.47 | 0,0572 | prostaglandin D2 synthase 21kDa (brain) |
|  | *PTGER1* | 2.93 | 2.95 | -1.01 | 0,2085 | prostaglandin E receptor 1 |
|  | *PTGER2* | 3.28 | 3.8 | -1.43 | 0,3486 | prostaglandin E receptor 2 |
|  | *PTGER3* | 2.59 | 2.22 | 1.29 | 0,0879 | prostaglandin E receptor 3 (subtype EP3) |
|  | *PTGER4* | 8.17 | 4.59 | 11.94 | 0,0203 | prostaglandin E receptor 4 (subtype EP4) |
|  | *PTGES* | 2.88 | 4.03 | -2.21 | 0,3065 | prostaglandin E synthase |
|  | *PTGES2* | 3.74 | 4.62 | -1.83 | 0,098 | prostaglandin E synthase 2 |
|  | *PTGES3* | 7.52 | 6.62 | 1.87 | 0,0331 | prostaglandin E synthase 3 (cytosolic) |
|  | *PTGFR* | 2.7 | 2.29 | 1.33 | 0,8367 | prostaglandin F receptor (FP) |
|  | *PTGFRN* | 2.4 | 2.38 | 1.01 | 0,9562 | prostaglandin F2 receptor inhibitor |
|  | *PTGIR* | 3.42 | 3.31 | 1.08 | 0,7848 | prostaglandin I2 (prostacyclin) receptor (IP) |
|  | *PTGIS* | 2.96 | 3.1 | -1.1 | 0,3038 | prostaglandin I2 (prostacyclin) synthase |
|  | *PTGR1* | 2.05 | 2.15 | -1.07 | 0,377 | prostaglandin reductase 1 |
|  | *PTGR2* | 2.3 | 2.17 | 1.1 | 0,7705 | prostaglandin reductase 2 |
|  | *PTGS1* | 5.43 | 6.15 | -1.65 | 0,5676 | prostaglandin-endoperoxide synthase 1 (prostaglandin G/H synthase and cyclooxygenase) |
|  | *PTGS2* | 7.42 | 6.2 | 2.32 | 0,5588 | prostaglandin-endoperoxide synthase 2 (prostaglandin G/H synthase and cyclooxygenase) |
